# Pharmacological and non-pharmacological interventions for adults with ADHD: protocol for a systematic review and network meta-analysis

**DOI:** 10.1136/bmjopen-2021-058102

**Published:** 2022-03-11

**Authors:** Samuele Cortese, Cinzia Del Giovane, Samuel Chamberlain, Alexandra Philipsen, Susan Young, Andrea Bilbow, Andrea Cipriani

**Affiliations:** 1 School of Psychology, Centre for Innovation in Mental Health (CIMH), Faculty of Environmental and Life Sciences, University of Southampton, Southampton, UK; 2 Clinical and Experimental Sciences (CNS and Psychiatry), Faculty of Medicine, University of Southampton, Southampton, UK; 3 Solent NHS Trust, Southampton, UK; 4 Hassenfeld Children’s Hospital at NYU Langone, New York University Child Study Center, New York City, New York, USA; 5 Division of Psychiatry and Applied Psychology, School of Medicine, University of Nottingham, Nottingham, UK; 6 Department of Medical and Surgical Sciences for Children and Adults, University of Modena and Reggio, Modena, Italy; 7 Institute of Primary Health Care, University of Bern, Bern, Switzerland; 8 Department of Psychiatry, Faculty of Medicine, University of Southampton, Southampton, UK; 9 Southern Health NHS Foundation Trust, Southampton, UK; 10 Department of Psychiatry and Psychotherapy, Rheinische Friedrich-Wilhelms-Universitat Bonn, Bonn, Germany; 11 Department of Psychology, Reykjavik University, Reykjavik, Iceland; 12 Attention Deficit Disorder Information and Support Service, London, UK; 13 ADHD-Europe, Brussels, Belgium; 14 Department of Psychiatry, University of Oxford, Oxford, UK; 15 Oxford Health NHS Foundation Trust, Oxford, UK

**Keywords:** adult psychiatry, impulse control disorders, clinical trials

## Abstract

**Introduction:**

It is unclear how pharmacological and non-pharmacological interventions compare with each other in terms of efficacy and tolerability for core symptoms and additional problems in adults with attention-deficit/hyperactivity disorder (ADHD). We aim to conduct the first network meta-analysis (NMA) comparing pharmacological and non-pharmacological interventions (or their combinations) in adults with ADHD.

**Methods and analysis:**

We will follow the Preferred Reporting Items for Systematic Reviews and Meta-Analyses (PRISMA) statement for NMAs. We will search a broad set of electronic databases/registries and contact drug companies and experts in the field to retrieve published and unpublished randomised controlled trials (RCTs) (parallel or cross-over) of medications (either licensed or unlicensed) and any non-pharmacological intervention in adults (≥18 years) with ADHD. Primary outcomes will be: (1) change in severity of ADHD core symptoms, and (2) acceptability (all-cause discontinuation). Secondary outcomes will include tolerability (drop-out due to side effects) and change in the severity of emotional dysregulation, executive dysfunctions and quality of life. The risk of bias in each individual RCT included in the NMA will be assessed using the Cochrane Risk of Bias tool-version 2. We will evaluate the transitivity assumption comparing the distribution of possible effect modifiers across treatment comparisons. We will perform Bayesian NMA for each outcome with random-effects model in OpenBUGS. Pooled estimates of NMA will be obtained using the Markov Chains Monte Carlo method. We will judge the credibility in the evidence derived from the NMA using the CINeMA tool (which includes assessment of publication bias). We will conduct a series of sensitivity analyses to assess the robustness of the findings.

**Ethics and dissemination:**

As this is the protocol for an aggregate-data level NMA, ethical approval will not be required. Results will be disseminated at national/international conferences and in peer-reviewed journals.

**PROSPERO registration number:**

CRD42021265576.

**Strengths and limitations of this study**

- The study will be conducted by a team with extensive expertise in the clinical assessment and treatment of attention-deficit/hyperactivity disorder (ADHD), as well as in advanced network meta-analysis (NMA) statistics.
- The protocol was designed and the study will be carried out with the involvement of patients and members of the public in the review team.
- We will include both published and unpublished data, systematically gathered by drug manufacturers and study authors.
- We will include, as outcomes, both ADHD core symptoms and related clinical problems, thus increasing the ecological validity of the study.
- The main limitation is that the proposed NMA will include aggregate-level data, rather than individual patient level data.

## Introduction

Attention-deficit/hyperactivity disorder (ADHD) is the most common neurodevelopmental condition in children,^1 2^ and its impairing symptoms persist into adulthood in up to ~75% of childhood cases.^3 4^ Adult ADHD has a prevalence estimated at ~2.5%^5^ and is commonly comorbid with other disorders (eg, depression or anxiety)^6^ and with problems such as emotional dysregulation, which are often the main trigger for a referral to clinical services.^6^ If untreated, adult ADHD is associated with substantial societal burden, including significantly increased risk of unemployment, substance abuse, criminal acts, accidents and mortality.^6^ The personal and societal costs of untreated ADHD in adults are estimated at around £20 000/person/year.^7^

Both pharmacological and non-pharmacological (eg, psychological) treatments are available for adults with ADHD.^8^ Pharmacological and non-pharmacological interventions should be considered to be complementary, rather than mutually exclusive options. For instance, while stimulants are considered highly effective in decreasing the severity of adult ADHD core symptoms over the short-medium term (effect size, ES: ~0.8),^9^ their efficacy in the treatment of emotional dysregulation is lower (ES: ~0.3–0.5),^10^ suggesting the need for additional pharmacological or non-pharmacological options.

The current ADHD guidelines from the National Institute for Health and Care Excellence (NICE) recommend pharmacotherapy (stimulants followed by the selective norepinephrine reuptake inhibitor atomoxetine) as first-line treatment options for adult ADHD, with psychological therapies as second option.^11^ However, the recommendation on the sequencing of pharmacological and non-pharmacological options was based on one randomised clinical trial (RCT) only,^12^ comparing head-to-head pharmacotherapy and psychological treatment, retrieved from a literature search (up to 28 April 2017) that is now outdated. Since the NICE guidelines were published, a number of RCTs have been published pointing to significant efficacy and good tolerability of a variety of non-pharmacological interventions—including cognitive behavioural therapy, dialectic behavioural therapy, mindfulness, cognitive training and neurofeedback—for ADHD core symptoms and/or associated dysfunctions.^13^ Additionally, due to the paucity of RCTs, the NICE committee was not able to make any evidence-based recommendation on which type(s) of non-pharmacological treatments are preferred. This is highly problematic in particular for those patients who do not opt for or are unable to tolerate a pharmacologic treatment and need to be informed on the comparative efficacy/tolerability of currently available non-pharmacologic options. Furthermore, recent studies have assessed internet delivered non-pharmacological interventions, to possibly maximise efficiency and cost-effectiveness (eg, 14 15). This is particularly relevant considering the need for remote assessment/treatment prompted by the current pandemic-related restrictions and the likely push towards digital interventions in the post-COVID-19 era.

Therefore, there is a need for updated evidence synthesis regarding how non-pharmacological interventions—and different ways to deliver them —compare with each other, to pharmacologic treatments or to combinations of pharmacological and non-pharmacological interventions in terms of efficacy and tolerability on specific relevant outcomes (eg, ADHD core symptoms, emotional dysregulation and executive functions) in adults with ADHD.

A well-powered RCT would be suited to compare pharmacological and non-pharmacological options, but there are obvious financial and logistic constraints in conducting well-powered RCTs comparing all interventions for ADHD in adults. Network meta-analysis (NMA), which allows for the comparison of two or more interventions even when they have not been compared head-to-head in the studies included in the meta-analysis,^16^ provides a cost-effective, practical option to address this gap.

A scoping search in PubMed/Medline (PubMed Central and Europe PubMed Central), PsycInfo and Embase (up to 1 October 2021) using search terms for ‘ADHD’, ‘adults’ and ‘network meta-analysis’ did not find any NMA including, in the same network, pharmacologic and non-pharmacologic interventions for adults with ADHD. Based on our searches, no protocol for such NMA has been registered in PROSPERO or other registries at the time of writing.

Therefore, we aim to conduct the first systematic review/NMA of published and unpublished RCTs to assess the comparative efficacy and tolerability of UK licensed and unlicensed medications for ADHD, non-pharmacological treatments, or their combination, on ADHD core symptoms severity and related dysfunctions (eg, emotional dysregulation) in adults with ADHD.

## Methods and analysis

The methods of the proposed study are based on the Preferred Reporting Items for Systematic Reviews and Meta-Analyses (PRISMA) statement and its extension for NMA.^17^ The methods are in line with those of another NMA^18^ of pharmacological and non-pharmacological interventions for depressive disorder.

The protocol of the present NMA is preregistered in PROSPERO.^19^

We plan to start the study on 1 March 2022 and to complete it by 1 March 2024.

### Search

We will update the search for RCTs of medications for ADHD from an NMA^9^ published in 2018 and conduct a de novo search for the non-pharmacological interventions. The search will be conducted with the support of Systematic Review Solutions Ltd. (SRS), an independent health research service company specialising in evidence-based medicine methods and meta-research training, production of systematic reviews and Health Technology Assessment reports, and development of clinical practice guidelines. SRS conducted the search for the previous NMA.^9^ Using a similar search strategy, we will search a broad set of electronic databases, including PubMed, BIOSIS Previews, CINAHL, the Cochrane Central Register of Controlled Trials, EMBASE, ERIC, MEDLINE, PsycINFO, OpenGrey, Web of Science Core Collection, ProQuest Dissertations and Theses (UK and Ireland), ProQuest Dissertations and Theses and the WHO International Trials Registry Platform, including *ClinicalTrials.gov*, with no language/type of document restrictions. For the specific syntax for each database, see online supplemental 1.

10.1136/bmjopen-2021-058102.supp1 Supplementary data

We will also search the US Food and Drug Administration, European Medicines Agency and relevant drug manufacturers’ websites for RCTs of medications. We will also endeavour to gather relevant unpublished data for pharmacological and non-pharmacological interventions by contacting drug companies, study authors and the members of key scientific organisations of ADHD worldwide. Specifically, we will contact the European Network of Hyperkinetic disorder (Eunethydis), the World ADHD Federation, the he American Professional Society of ADHD and Related Disorders (APSARD) and the Canadian ADHD Resource Alliance (CADDRA) to advertise the study and query about the existence of any eligible unpublished study.

### Selection criteria

#### Study design

We will include parallel or cross-over RCTs of at least 1-week duration for pharmacological treatment, in line with prior work,^9^ and of at least four sessions for psychotherapy. For cross-over studies of medications, to address concerns around possible ‘carry over’ effects, we will use data from the precross-over phase, when reported in the paper. When data for the precross-over phase are not reported, we will contact study authors to gather them. If precross-over data are not reported and not available on request, we will use data at the end point (after crossing over), only if there was a washout period (as reported in Cortese *et al*)^9^ between the two phases (precross-over and postcross-over) of the RCT. For trials of neurotherapies (eg, neurofeedback), we will include RCTs of any length deemed appropriate for these approaches. For trials of medications, cognitive training or neurotherapies alone, we will include only double-blind RCTs (patients and raters blinded). For trials of psychotherapy alone or the combination of medications and psychotherapy, we will include trials in which observers and/or raters were masked and/or participants were assessed by self-rating ADHD scales, because participants and therapists cannot be blinded, but we will then conduct a sensitivity analysis including only double blind RCTs (please see below). We will exclude studies with enrichment designs (eg, trials selecting responders only after a run-in phase), because these types of trial can potentially inflate efficacy and tolerability estimates.^20^

#### Participants

We will retain RCTs including adults (≥18 years) with a formal diagnosis of ADHD according to DSM-III, DSM III-R, DSM-IV (TR), DSM-5, ICD-9, ICD-10 or ICD-11. We will not restrict our search by ADHD subtype or presentation, sex, ethnicity, IQ, socioeconomic status or comorbidities.

#### Interventions

As in prior work,^9^ pharmacological interventions will include stimulants (methylphenidate and amphetamines, including lisdexamphetamine); atomoxetine; guanfacine XR, clonidine, bupropion and modafinil. We will also search for eligible studies of viloxazine, which has been recently approved by the FDA for children and adolescents (aged 6–17) with ADHD.^21^ In the analyses, we will lump methylphenidate and amphetamines as: (1) a previous NMA^9^ did not find any significant difference, in terms of efficacy, between methylphenidate and amphetamines in adults with ADHD; (2) accordingly, current NICE guidelines^11^ recommend methylphenidate *or* lisdexamphetamine (or other amphetamines) as first-line pharmacological treatment for adults. Any type of non-pharmacological intervention will be considered.

#### Controls

The pharmacological control condition will be a pill placebo; non-pharmacological controls will include waiting list, treatment as usual, clinical management, active control in psychotherapy, and psychological placebo (sham).

#### Outcomes

Primary outcomes: (1) change in severity of ADHD core symptoms, according to a standardised rating scale.^9^ We will consider separately self-rated ADHD core symptoms and observer as well as clinician-rated symptoms; (2) acceptability (all-cause discontinuation measured by the proportion of patients who withdrew from the study for any reason). Secondary outcomes: tolerability (drop-out due to side effects); change in the severity of emotional dysregulation, measured with any of the scales listed in Lenzi *et al*;^10^ executive dysfunctions, based on any of the scales in Tamminga *et al*,^22^ and quality of life, measured with any of the scales listed in Tsujii *et al*.^23^

### Data collection

We will select studies, and extract/collect data in a two-step process. First, two independent investigators will screen the titles and abstracts we identified. Second, two independent investigators will obtain and read the full texts of all potentially relevant studies and determine the final list of studies to include. Any disagreement will be resolved by senior investigators. We will extract data into prespecified data extraction forms.^24^ For each study, we will extract information on study characteristics (eg, setting, study design and sample size), participant characteristics (eg, mean age and range, presence of comorbidities and concomitant therapies), interventions and controls (eg, dose, frequency of treatment) and outcomes. We will systematically contact study when needed to gather unpublished information/data.

### Study risk of bias assessment

The risk of bias in each individual RCT included in our NMA will be assessed using the Cochrane Risk of Bias tool-version 2 (RoB-2), as recommended in The Cochrane Handbook of Systematic Reviews of Interventions.^25^ The tool includes five domains through which bias might be introduced into the result. For individually randomised trials (including cross-over trials), these include:

1. Bias arising from the randomisation process.
2. Bias due to deviations from intended interventions.
3. Bias due to missing outcome data.
4. Bias in measurement of the outcome.
5. Bias in selection of the reported result.

We will use the appropriate templates for randomised parallel-group and cross-over trials, respectively.

### Measures of treatment effects

For continuous outcomes, we will use mean difference (MD) as a measurement of treatment effect, with the relative 95% CI, when studies assessed the outcome with the same instruments; standardised mean difference (SMD, Cohen’s d) when studies used different instruments.^26^ We will use published mean values and standard deviations (SDs). If SDs are not available, they will be estimated by conversion from SEs, p-values, CIs or t-values.^27^ If none of the above values is available from the published paper, we will contact the authors of the study to obtain information. If the information is not provided by the study author, we will employ a validated method for imputation to derive missing SDs.^28^

For dichotomous outcomes, we will calculate the OR and relative 95% CI. Missing dichotomous outcome data will be handled according to the intention-to-treat principle. Participants who drop out after randomisation will be considered as having a negative outcome.

### Assessment of clinical and methodological heterogeneity within treatment comparisons

In each pairwise comparison, patient characteristics, treatments and outcome deﬁnitions of included studies should be similar.^26^ We will produce descriptive statistics for studies and study population characteristics across included trials to assess clinical and methodological heterogeneity. Within each pairwise comparison, we will compare these characteristics to assess the presence of clinical heterogeneity.

### Assessment of transitivity across treatment comparisons

It is appropriate to use NMA if the assumption of transitivity can be defended. Transitivity holds when the distributions of the potential effect modifiers, like study and patient-level covariates, are balanced across all pairwise comparisons.^29 30^ To assess the transitivity assumption, we will compare the distribution of clinical and methodological variables (eg, ADHD severity at baseline, comorbidities, adherence and treatment duration) that could act as effect modifiers across treatment comparisons.

### Data analysis

First, we will conduct conventional pairwise meta-analyses with a random-effects model in STATA V.16.1 for all outcomes and comparisons with at least two studies. Then, we will perform Bayesian NMA for each outcome with random-effects model in OpenBUGS,^31^ accounting for correlations induced by multiarm studies.^32^ Pooled estimates of NMA will be obtained using the Markov Chains Monte Carlo method. We will employ the binomial (dichotomous outcomes) and normal (continuous outcomes) likelihood functions and will use vague prior distributions for the treatment effects and a minimally informative prior distribution for the common heterogeneity SD depending on the outcome. We will examine Gelman-Rubin trace plots to check that multiple chains achieve convergence. All results will be reported as treatment effects (MD, SMD or OR) and their 95% credible intervals. NMA results will be presented in league tables and forest plots.^33^

We will estimate heterogeneity variances for each pairwise comparison in standard pairwise meta-analyses and assess the presence of statistical heterogeneity by visually inspecting the forest plots and calculating the I-squared statistic.^34^ In the NMA, we will assume a common estimate for heterogeneity variance across comparisons and base our assessment of statistical heterogeneity in the whole network by comparing the magnitude of the common heterogeneity variance (τ2) with the empirical distribution as derived by Rhodes and Turner.^35 36^ We will also calculate the total I-squared statistic.

Statistical disagreement between direct and indirect effect sizes (incoherence) will be evaluated globally, by comparison of the fit and parsimony of consistency and inconsistency models, and locally, by calculation of the difference between direct and indirect estimates in all closed loops in the network.^37^ The node splitting method, which separates evidence on a particular comparison into direct and indirect evidence, will be used to calculate the inconsistency of the model.^38^ To determine whether the results are affected by possible effect modifiers, we will conduct network meta-regression and subgroup analysis according to the following variables: study sponsorship, treatment duration, comorbid psychiatric disorders, study risk of bias, mean baseline severity and percentage of participants treated with stable doses of medications in non-pharmacological RCTs.

We will then use the Surface Under the Cumulative RAnking (SUCRA) curve to measure, for any outcome, the probability each treatment is the best option among all treatments included in the network treatment and express the SUCRA measure as a percentage.^39^ We will use a comparison-adjusted funnel plot for active treatments versus control to determine the possibility of small study-effects.^33 40^

We will assess the certainty of evidence derived using CINeMA (http://cinema.ispm.ch/).^41 42^ CINeMA is a software which uses the netmeta R-package for performing NMA of the data. The tool considers the following domains: within-study bias, publication bias, indirectness, imprecision, heterogeneity and incoherence. It classifies overall confidence in evidence for each comparison as high, moderate, low or very low. In particular, for publication bias, we will use the new tool implemented within CINeMA, ROB-MEN, that allow to evaluate the impact of this bias on the results of NMA of interventions.^43^

We will finally conduct sensitivity analyses for primary outcomes by excluding trials without unpublished data, trials with imputed data, trials with overall sample size smaller than 20 and trials with non-blinded assessments. We will also conduct a sensitivity analysis combing guanfacine and clonidine in the same node.

### Patient and public involvement

Our patient and public involvement coauthor, Ms. Bilbow, CEO of The National Attention Deficit Disorder Information and Support Service (ADDISS), one of the largest UK charities of patients with ADHD, has played a central role in the development of this protocol since its initial design. During the preparation of the present proposal, Ms. Bilbow: liaised with representatives (patients) of ADDISS, and ADHD Europe to gather their feedback on the proposal; based on the feedback form patients, critically commented on the overarching plan of the application, highlighting that it covers an important gap perceived as crucial by patients with ADHD and their families; noted the importance of comparing different types of non-pharmacological interventions given the patchy provision across the UK and uncertainties around the evidence base supporting at least some types of non-pharmacological approaches; recommended inclusion of quality of life as a secondary outcome measure.

As this is a protocol, no patients were directly involved in this study.

## Ethics and dissemination

### Ethics

As this is the protocol of an aggregate-data level NMA, no ethical approval will be needed.

### Dissemination

On publication, the full data set of the NMA and the codes for the analyses will be available online freely in Mendeley Data, a secure online repository for research data. The results of the study will be disseminated nationally, via conferences organised by groups of people with lived experience (eg, National Attention Deficit Disorder Information and Support Service and ADHD Foundation) and professional organisations (eg, UKAAN, Royal College of Psychiatrists). Results will also be disseminated internationally via conferences (for service users: eg, annual meeting of ADHD Europe; for professionals, eg, meetings of the WFA, Eunethydis, APSARD) and publications in peer-reviewed journals in the field of psychiatry/psychology and general medicine.

## Supplementary Material

Reviewer comments

Author's manuscript

## References

[1] Cortese S, Solmi M, Arrondo G, et al. Association between mental disorders and somatic conditions: protocol for an umbrella review. Evid Based Ment Health 2020;23:135–9. 10.1136/ebmental-2020-300158 32900790 PMC10231570

[2] Barican JL, Yung D, Schwartz C, et al. Prevalence of childhood mental disorders in high-income countries: a systematic review and meta-analysis to inform policymaking. Evid Based Ment Health 2022;25:36–44. 10.1136/ebmental-2021-300277 34281985 PMC8788041

[3] Zhang L, Lagerberg T, Chen Q, et al. Prediction of treatment dosage and duration from free-text prescriptions: an application to ADHD medications in the Swedish prescribed drug register. Evid Based Ment Health 2021;24:146–52. 10.1136/ebmental-2020-300231 33795353 PMC8543229

[4] Sibley MH, Mitchell JT, Becker SP. Method of adult diagnosis influences estimated persistence of childhood ADHD: a systematic review of longitudinal studies. Lancet Psychiatry 2016;3:1157–65. 10.1016/S2215-0366(16)30190-0 27745869

[5] Song P, Zha M, Yang Q, et al. The prevalence of adult attention-deficit hyperactivity disorder: a global systematic review and meta-analysis. J Glob Health 2021;11:04009. 10.7189/jogh.11.04009 33692893 PMC7916320

[6] Faraone SV, Asherson P, Banaschewski T, et al. Attention-deficit/hyperactivity disorder. Nat Rev Dis Primers 2015;1:15020. 10.1038/nrdp.2015.20 27189265

[7] Daley D, Jacobsen RH, Lange A-M, et al. The economic burden of adult attention deficit hyperactivity disorder: a sibling comparison cost analysis. Eur Psychiatry 2019;61:41–8. 10.1016/j.eurpsy.2019.06.011 31288209

[8] Cortese S. Pharmacologic treatment of attention deficit-hyperactivity disorder. N Engl J Med 2020;383:1050–6. 10.1056/NEJMra1917069 32905677

[9] Cortese S, Adamo N, Del Giovane C, et al. Comparative efficacy and tolerability of medications for attention-deficit hyperactivity disorder in children, adolescents, and adults: a systematic review and network meta-analysis. Lancet Psychiatry 2018;5:727–38. 10.1016/S2215-0366(18)30269-4 30097390 PMC6109107

[10] Lenzi F, Cortese S, Harris J, et al. Pharmacotherapy of emotional dysregulation in adults with ADHD: a systematic review and meta-analysis. Neurosci Biobehav Rev 2018;84:359–67. 10.1016/j.neubiorev.2017.08.010 28837827

[11] National Institute for Health and Care Excellence. Attention deficit hyperactivity disorder: diagnosis and management, 2018. Available: https://www.nice.org.uk/guidance/ng87 29634174

[12] Philipsen A, Jans T, Graf E, et al. Effects of group psychotherapy, individual counseling, methylphenidate, and placebo in the treatment of adult attention-deficit/hyperactivity disorder: a randomized clinical trial. JAMA Psychiatry 2015;72:1199–210. 10.1001/jamapsychiatry.2015.2146 26536057

[13] Nimmo-Smith V, Merwood A, Hank D, et al. Non-pharmacological interventions for adult ADHD: a systematic review. Psychol Med 2020;50:529–41. 10.1017/S0033291720000069 32036811

[14] Moëll B, Kollberg L, Nasri B, et al. Living SMART — a randomized controlled trial of a guided online course teaching adults with ADHD or sub-clinical ADHD to use smartphones to structure their everyday life. Internet Interv 2015;2:24–31. 10.1016/j.invent.2014.11.004

[15] Pettersson R, Söderström S, Edlund-Söderström K, et al. Internet-based cognitive behavioral therapy for adults with ADHD in outpatient psychiatric care. J Atten Disord 2017;21:508–21. 10.1177/1087054714539998 24970720

[16] Xiang Y, Cipriani A, Teng T, et al. Comparative efficacy and acceptability of psychotherapies for post-traumatic stress disorder in children and adolescents: a systematic review and network meta-analysis. Evid Based Ment Health 2021;24:153–60. 10.1136/ebmental-2021-300346 34599050 PMC8543231

[17] Hutton B, Salanti G, Caldwell DM, et al. The PRISMA extension statement for reporting of systematic reviews incorporating network meta-analyses of health care interventions: checklist and explanations. Ann Intern Med 2015;162:777–84. 10.7326/M14-2385 26030634

[18] Zhou X, Teng T, Zhang Y, et al. Comparative efficacy and acceptability of antidepressants, psychotherapies, and their combination for acute treatment of children and adolescents with depressive disorder: a systematic review and network meta-analysis. Lancet Psychiatry 2020;7:581–601. 10.1016/S2215-0366(20)30137-1 32563306 PMC7303954

[19] National Institute for Health Research. Available: https://www.crd.york.ac.uk/PROSPERO/display_record.php?RecordID=265576

[20] Wong ICK, Banaschewski T, Buitelaar J, et al. Emerging challenges in pharmacotherapy research on attention-deficit hyperactivity disorder-outcome measures beyond symptom control and clinical trials. Lancet Psychiatry 2019;6:528–37. 10.1016/S2215-0366(19)30096-3 31122482

[21] Findling RL, Candler SA, Nasser AF, et al. Viloxazine in the management of CNS disorders: a historical overview and current status. CNS Drugs 2021;35:643–53. 10.1007/s40263-021-00825-w 34003459 PMC8219567

[22] Tamminga HGH, Reneman L, Huizenga HM, et al. Effects of methylphenidate on executive functioning in attention-deficit/hyperactivity disorder across the lifespan: a meta-regression analysis. Psychol Med 2016;46:1791–807. 10.1017/S0033291716000350 27019103

[23] Tsujii N, Okada T, Usami M, et al. Effect of continuing and discontinuing medications on quality of life after symptomatic remission in attention-deficit/hyperactivity disorder: a systematic review and meta-analysis. J Clin Psychiatry 2020;81. 10.4088/JCP.19r13015. [Epub ahead of print: 24 03 2020]. 32237294

[24] Pedder H, Sarri G, Keeney E, et al. Data extraction for complex meta-analysis (DECiMAL) guide. Syst Rev 2016;5:212. 10.1186/s13643-016-0368-4 27964742 PMC5154138

[25] Higgins JT, Chandler J, Cumpston M. Cochrane Handbook for systematic reviews of interventions version 6.2 (updated February 2021), 2021. Cochrane. Available: www.training.cochrane.org/handbook

[26] Solmi M, Wade TD, Byrne S, et al. Comparative efficacy and acceptability of psychological interventions for the treatment of adult outpatients with anorexia nervosa: a systematic review and network meta-analysis. Lancet Psychiatry 2021;8:215–24. 10.1016/S2215-0366(20)30566-6 33600749

[27] Altman DG, Bland JM. Detecting skewness from summary information. BMJ 1996;313:1200. 10.1136/bmj.313.7066.1200 PMC2352469 8916759

[28] Furukawa TA, Barbui C, Cipriani A, et al. Imputing missing standard deviations in meta-analyses can provide accurate results. J Clin Epidemiol 2006;59:7–10. 10.1016/j.jclinepi.2005.06.006 16360555

[29] Cipriani A, Higgins JPT, Geddes JR, et al. Conceptual and technical challenges in network meta-analysis. Ann Intern Med 2013;159:130–7. 10.7326/0003-4819-159-2-201307160-00008 23856683

[30] Salanti G. Indirect and mixed-treatment comparison, network, or multiple-treatments meta-analysis: many names, many benefits, many concerns for the next generation evidence synthesis tool. Res Synth Methods 2012;3:80–97. 10.1002/jrsm.1037 26062083

[31] Lunn D, Spiegelhalter D, Thomas A, et al. The BUGS project: evolution, critique and future directions. Stat Med 2009;28:3049–67. 10.1002/sim.3680 19630097

[32] Lumley T. Network meta-analysis for indirect treatment comparisons. Stat Med 2002;21:2313–24. 10.1002/sim.1201 12210616

[33] Chaimani A, Higgins JPT, Mavridis D, et al. Graphical tools for network meta-analysis in STATA. PLoS One 2013;8:e76654. 10.1371/journal.pone.0076654 24098547 PMC3789683

[34] Higgins JPT, Thompson SG, Deeks JJ, et al. Measuring inconsistency in meta-analyses. BMJ 2003;327:557–60. 10.1136/bmj.327.7414.557 12958120 PMC192859

[35] Rhodes KM, Turner RM, Higgins JPT. Predictive distributions were developed for the extent of heterogeneity in meta-analyses of continuous outcome data. J Clin Epidemiol 2015;68:52–60. 10.1016/j.jclinepi.2014.08.012 25304503 PMC4270451

[36] Turner RM, Davey J, Clarke MJ, et al. Predicting the extent of heterogeneity in meta-analysis, using empirical data from the Cochrane database of systematic reviews. Int J Epidemiol 2012;41:818–27. 10.1093/ije/dys041 22461129 PMC3396310

[37] Veroniki AA, Vasiliadis HS, Higgins JPT, et al. Evaluation of inconsistency in networks of interventions. Int J Epidemiol 2013;42:332–45. 10.1093/ije/dys222 23508418 PMC5411010

[38] Higgins JPT, Jackson D, Barrett JK, et al. Consistency and inconsistency in network meta-analysis: concepts and models for multi-arm studies. Res Synth Methods 2012;3:98–110. 10.1002/jrsm.1044 26062084 PMC4433772

[39] Salanti G, Ades AE, Ioannidis JPA. Graphical methods and numerical summaries for presenting results from multiple-treatment meta-analysis: an overview and tutorial. J Clin Epidemiol 2011;64:163–71. 10.1016/j.jclinepi.2010.03.016 20688472

[40] Peters JL, Sutton AJ, Jones DR, et al. Contour-enhanced meta-analysis funnel plots help distinguish publication bias from other causes of asymmetry. J Clin Epidemiol 2008;61:991–6. 10.1016/j.jclinepi.2007.11.010 18538991

[41] Salanti G, Del Giovane C, Chaimani A, et al. Evaluating the quality of evidence from a network meta-analysis. PLoS One 2014;9:e99682. 10.1371/journal.pone.0099682 24992266 PMC4084629

[42] Nikolakopoulou A, Higgins JPT, Papakonstantinou T, et al. CINeMA: an approach for assessing confidence in the results of a network meta-analysis. PLoS Med 2020;17:e1003082. 10.1371/journal.pmed.1003082 32243458 PMC7122720

[43] Chiocchia V, Nikolakopoulou A, Higgins JPT, et al. ROB-MEN: a tool to assess risk of bias due to missing evidence in network meta-analysis. BMC Med 2021;19:304. 10.1186/s12916-021-02166-3 34809639 PMC8609747

