# Supplementary material for: Pharmacological and non-pharmacological interventions for adults with ADHD: protocol for a systematic review and network meta-analysis
Source: BMJ Open. 2022 Mar 11;12(3):e058102. doi: 10.1136/bmjopen-2021-058102 (PMC8919448; doi:10.1136/bmjopen-2021-058102)
Supplement: Supplementary data [file bmjopen-2021-058102supp001.pdf]

Search syntax for each database (in alphabetical order):

A. BIOSIS Previews
TOPIC: (adhd OR hkd OR addh OR hyperkine* OR "attention deficit*" OR hyper-activ* OR hyperactiv* OR overactive OR inattentive OR impulsiv*) AND **TOPIC:** AND **TOPIC:** (RCT OR ((clinical OR control*) NEAR/10 trial*) OR crossover OR "cross over" OR cross-over OR randomi* OR (random* NEAR/1 (allocat* OR assign* OR select*)) OR blind* OR placebo OR "control group")
Indexes=BIOSIS Previews Timespan=All years

B. EMBASE
1. exp Attention Deficit Disorder with Hyperactivity/ or (adhd or hkd or addh or hyperkine* or "attention deficit*" or hyper-activ* or hyperactiv* or overactive or inattentive or impulsiv*).ti,ab.
2. (random$ or factorial$ or crossover$ or (cross over$) or cross-over$ or placebo$ or (doubl$ adj blind$) or (singl$ adj blind$) or assign$ or allocat$ or volunteer$).mp. or crossover-procedure/ or double-blind procedure/ or randomized controlled trial/ or single-blind procedure/
3. limit 2 to human
4. 1 and 2 and 3
No limitations

C. ERIC
((SU.EXACT.EXPLODE("Attention Deficit Disorders") OR ti(adhd OR hkd OR addh OR hyperkine* OR "attention deficit*" OR hyper-activ* OR hyperactiv* OR overactive OR inattentive OR impulsiv*) OR ab(adhd OR hkd OR addh OR hyperkine* OR "attention deficit*" OR hyper-activ* OR hyperactiv* OR overactive OR inattentive OR impulsiv*)) AND (ti(RCT OR ((clinical OR control*) NEAR/10 trial*) OR crossover OR "cross over" OR cross-over OR randomi* OR (random* NEAR/1 (allocat* OR assign* OR select*)) OR blind* OR placebo OR "control group") OR ab(RCT OR ((clinical OR control*) NEAR/10 trial*) OR crossover OR "cross over" OR cross-over OR randomi* OR (random* NEAR/1 (allocat* OR assign* OR select*)) OR blind* OR placebo OR "control group"))
No limitations

D. International Clinical Trials Registry Platform (WHO ICTRP)
(adhd OR hkd OR addh OR hyperkine* OR "attention deficit*" OR hyper-activ* OR hyperactiv* OR overactive OR inattentive OR impulsiv*) in Condition Field AND

E. MEDLINE
1. exp Attention Deficit Disorder with Hyperactivity/ or (adhd or hkd or addh or hyperkine* or "attention deficit*" or hyper-activ* or hyperactiv* or overactive or inattentive or impulsiv*).ti,ab.
2. (randomized controlled trial or controlled clinical trial).pt. or random$.ab. or placebo.ab. or drug therapy.fs. or trial.ab. or groups.ab.
3. exp animals/ not humans.sh.
4. 2 not 3
5. 1 and 2 and 4
No limitations

F. ProQuest Dissertations & Theses: UK & Ireland and ProQuest Dissertations & Theses A&I
((ti(adhd OR hkd OR addh OR hyperkine* OR "attention deficit*" OR hyper-activ* OR hyperactiv* OR overactive OR inattentive OR impulsiv*) OR ab(adhd OR hkd OR addh OR hyperkine* OR "attention deficit*" OR hyper-activ* OR hyperactiv* OR overactive OR inattentive OR impulsiv*)) AND (ti(RCT OR ((clinical OR control*) NEAR/10 trial*) OR crossover OR "cross over" OR cross-over OR randomi* OR (random* NEAR/1 (allocat* OR assign* OR select*)) OR blind* OR placebo OR "control group") OR ab(RCT OR ((clinical OR control*) NEAR/10 trial*) OR crossover OR "cross over" OR cross-over OR randomi* OR (random* NEAR/1 (allocat* OR assign* OR select*)) OR blind* OR placebo OR "control group"))
No limitations

G. PsycINFO
1. exp Attention Deficit Disorder with Hyperactivity/ or (adhd or hkd or addh or hyperkine* or "attention deficit*" or hyper-activ* or hyperactiv* or overactive or inattentive or impulsiv*).ti,ab.

2. (double-blind or random* assigned or control).tw.
3. and/1-2
4. limit 3 to human

No limitations

H. PubMed

("Attention Deficit Disorder with Hyperactivity"[Mesh] OR adhd[tiab] OR hkd[tiab] OR addh[tiab] OR hyperkine*[tiab] OR "attention deficit*"[tiab] OR hyper-activ*[tiab] OR hyperactiv*[tiab] OR overactive[tiab] OR inattentive[tiab] OR impulsiv*[tiab]) AND (randomized controlled trial[pt] OR controlled clinical trial[pt] OR randomized[tiab] OR placebo[tiab] OR clinical trials as topic[mesh:noexp] OR randomly[tiab] OR trial[ti]) NOT (animals[mh] NOT humans[mh])
Filter : adult (19+)

I. SIGLE

(adhd OR hkd OR addh OR hyperkine* OR "attention deficit*" OR hyper-activ* OR hyperactiv* OR overactive OR inattentive OR impulsiv*)

J. Cochrane Library

#1 MeSH descriptor: [Attention Deficit Disorder with Hyperactivity] explode all trees
#2 (adhd or hkd or addh or hyperkine* or "attention deficit*" or hyper-activ* or hyperactiv* or overactive or inattentive or impulsiv*):ti,ab
No limitations

K. Web of Science

TOPIC: (adhd OR hkd OR addh OR hyperkine* OR "attention deficit*" OR hyper-activ* OR hyperactiv* OR overactive OR inattentive OR impulsiv*) AND **TOPIC:** (RCT OR ((clinical OR control*) NEAR/10 trial*) OR crossover OR "cross over" OR cross-over OR randomi* OR (random* NEAR/1 (allocat* OR assign* OR select*)) OR blind* OR placebo OR "control group")
Indexes=SCI-EXPANDED, SSCI, CPCI-S, CPCI-SSH Timespan=All years
