# Supplementary material for: Pharmacological and non-pharmacological interventions for adults with ADHD: protocol for a systematic review and network meta-analysis
Source: BMJ Open. 2022 Mar 11;12(3):e058102. doi: 10.1136/bmjopen-2021-058102 (PMC8919448; doi:10.1136/bmjopen-2021-058102)
Supplement: Author's manuscript [file bmjopen-2021-058102.draft_revisions.pdf]

BMJ Open is committed to open peer review. As part of this commitment we make the peer review history of every article we publish publicly available.

When an article is published we post the peer reviewers' comments and the authors' responses online. We also post the versions of the paper that were used during peer review. These are the versions that the peer review comments apply to.

The versions of the paper that follow are the versions that were submitted during the peer review process. They are not the versions of record or the final published versions. They should not be cited or distributed as the published version of this manuscript.

BMJ Open is an open access journal and the full, final, typeset and author-corrected version of record of the manuscript is available on our site with no access controls, subscription charges or pay-per-view fees (http://bmjopen.bmj.com).

If you have any questions on BMJ Open's open peer review process please email info.bmjopen@bmj.com

BMJ Open

Pharmacological and non-pharmacological interventions for adults with ADHD: protocol for a systematic review and network meta-analysis

Journal:	*BMJ Open*
Manuscript ID	bmjopen-2021-058102
Article Type:	Protocol
Date Submitted by the Author:	07-Oct-2021
Complete List of Authors:	Cortese, Samuele; University of Southampton<br>Del Giovane, Cinzia; University of Bern<br>Chamberlain, Samuel; University of Southampton<br>Philipsen, Alexandra; Rheinische Friedrich-Wilhelms-Universitat Bonn, Department of Psychiatry and Psychotherapy<br>Young, Susan; University of Reykjavik,<br>Bilbow, Andrea; Attention Deficit Disorder Information and Support Service<br>Cipriani, Andrea; University of Oxford, Department of Psychiatry
Keywords:	Adult psychiatry < PSYCHIATRY, Impulse control disorders < PSYCHIATRY, Clinical trials < THERAPEUTICS

SCHOLARONE™
Manuscripts

Pharmacological and non-pharmacological interventions for adults with ADHD: protocol for a systematic review and network meta-analysis

Samuele Cortese [1-5], Cinzia Del Giovane [6-7], Samuel R. Chamberlain [8-9], Alexandra Philipsen [10], Susan Young [11-12], Andre Bilbow [13-14], Andrea Cipriani [15-17]

[1] School of Psychology, Centre for Innovation in Mental Health (CIMH), Faculty of Environmental and Life Sciences, University of Southampton, Highfield Campus, UK. samuele.cortese@soton.ac.uk

[2]Clinical and Experimental Sciences (CNS and Psychiatry), Faculty of Medicine, University of Southampton, Southampton, UK.

[3]Solent NHS Trust, Southampton, UK.

[4]Hassenfeld Children's Hospital at NYU Langone, New York University Child Study Center, New York, USA.

[5]Division of Psychiatry and Applied Psychology, School of Medicine, University of Nottingham, Nottingham, UK.

[6] Institute of Primary Health Care (BIHAM), University of Bern, Bern, Switzerland. cinzia.delgiovane@biham.unibe.ch

[7]Population Health Laboratory, University of Fribourg, Fribourg, Switzerland

[8] Department of Psychiatry, Faculty of Medicine, University of Southampton, Southampton, UK. S.R.Chamberlain@soton.ac.uk

[9] Southern Health NHS Foundation Trust, Southampton, UK.

[10] Department of Psychiatry and Psychotherapy, University of Bonn, Bonn, Germany. Alexandra.Philipsen@ukbonn.de

[11] Department of Psychology, Reykjavik University, Reykjavik, Iceland. SuzyYoung@aol.com

[12] ADHD Foundation, Liverpool, UK

[13] Attention Deficit Disorder Information and Support Service (ADDISS), Edgware, UK. andrea@addiss.co.uk

[14] ADHD-Europe, Brussels, Belgium.

[15] Department of Psychiatry, University of Oxford, Oxford, UK.
andrea.cipriani@psych.ox.ac.uk

[16] Oxford Health NHS Foundation Trust, Warneford Hospital, Oxford, UK.

[17] Oxford Precision Psychiatry Lab, NIHR Oxford Health Biomedical Research Centre, Oxford, UK.

Word count (main text): 2697

Address correspondence to: Prof. Samuele Cortese, School of Psychology, Centre for Innovation in Mental Health (CIMH), Faculty of Environmental and Life Sciences, University of Southampton, Highfield Campus, Building 44, Southampton, SO17 1BJ, UK, Phone: +44 (0) 2380599645, E-mail: Samuele.cortese@gmail.com

ABSTRACT

Introduction: It is unclear how pharmacological and non-pharmacological interventions compare with each other in terms of efficacy and tolerability for core symptoms and additional problems in adults with Attention-Deficit/Hyperactivity Disorder (ADHD). We aim to conduct the first network meta-analysis (NMA) including, in the same network, randomised controlled trials (RCTs) of pharmacological and non-pharmacological interventions (or their combinations) in adults with ADHD.

Methods and analysis: We will follow the Preferred Reporting Items for Systematic Reviews and Meta-Analyses (PRISMA) statement and its extension for NMAs. We will search a broad set of electronic databases/registries and contact drug companies and experts in the field to retrieve published and unpublished RCTs (parallel or cross-over) of medications (UK licensed or unlicensed) and any non-pharmacological intervention in adults (< 18 years) with ADHD. Primary outcomes will be: 1) change in severity of ADHD core symptoms, and 2) acceptability (all-cause discontinuation). Secondary outcomes will include tolerability (drop-out due to side effects) and change in the severity of emotional dysregulation, executive dysfunctions, and quality of life. The risk of bias in each individual RCT included in the NMA will be assessed using the Cochrane Risk of Bias tool-version 2. We will evaluate the transitivity assumption comparing the distribution of possible effect modifiers across treatment comparisons. We will perform Bayesian network meta-analysis for each outcome with random-effects model in OpenBUGS. Pooled estimates of network meta-analysis will be obtained using the Markov Chains Monte Carlo method. We will judge the credibility in the evidence derived from the NMA using the CINeMA tool. We will conduct a series of sensitivity analyses to assess the robustness of the findings.

For peer review only - http://bmjopen.bmj.com/site/about/guidelines.xhtml

Ethics and dissemination: As this is the protocol for an aggregate-data level NMA, ethical approval will not be required. Results will be disseminated at national/international conferences and in peer-reviewed journals.

Registration: PROSPERO: [ANONYMISED].

Keywords: ADHD; Treatment; pharmacological; adults; network meta-analysis

For peer review only

STRENGTHS AND LIMITATIONS OF THIS STUDY

- The study to which the present protocol refers will be the first NMA comparing pharmacological treatments with a broad range of non-pharmacological interventions for adults with ADHD
- The study will be conducted by a team with extensive expertise in the clinical assessment and treatment of ADHD, as well as in advanced NMA statistics, complemented by expertise from patient and public involvement (PPI) team members
- We plan to include both published and unpublished data systematically gathered by drug manufacturers and study authors
- We will include, as outcomes, both ADHD core symptoms and additional problems, thus increasing the ecologic validity of the study
- The main limitation is that the proposed NMA will include aggregate-data level, rather than individual patient level data. As such, results will refer to group averages

For peer review only

INTRODUCTION

Attention-Deficit/Hyperactivity Disorder (ADHD) is the most common neurodevelopmental condition in children,[1,2] and its impairing symptoms persist into adulthood in up to ~75% of childhood cases.[3,4] Adult ADHD has a prevalence estimated at ~2.5%[5] and is commonly comorbid with other disorders (e.g., depression or anxiety[6]) and with problems such as emotional dysregulation, which are often the main trigger for a referral to clinical services.[6] If untreated, adult ADHD is associated with substantial societal burden, including significantly increased risk of unemployment, substance abuse, criminal acts, accidents, and mortality.[6] The personal and societal costs of untreated ADHD in adults are estimated at around £20,000/person/yr.[7]

Both pharmacological and non-pharmacological (e.g., psychological) treatments are available for adults with ADHD.[8] Pharmacological and non-pharmacological interventions should be considered to be complementary, rather than mutually exclusive options. For instance, whilst stimulants are considered highly effective in decreasing the severity of adult ADHD core symptoms over the short-medium term (effect size, ES: ~ 0.8),[9] their efficacy in the treatment of emotional dysregulation is lower (ES: ~ 0.3-0.5),[10] suggesting the need for additional pharmacological or non-pharmacological options.

The current ADHD guidelines from the National Institute for Health and Care Excellence (NICE) recommend pharmacotherapy (stimulants followed by the selective noradrenaline reuptake inhibitor atomoxetine) as first-line treatment options for adult ADHD, with psychological therapies as second option.[11] However, the recommendation on the sequencing of pharmacological and non-pharmacological options was based on one randomised clinical trial (RCT) only,[12] comparing head-to-head pharmacotherapy and psychological treatment, retrieved from a literature search (up to 28 April 2017) that is now outdated. Since the NICE guidelines were published, a number of RCTs have been published pointing to significant

For peer review only

efficacy and good tolerability of a variety of non-pharmacological interventions - including cognitive behavioural therapy, dialectic behavioural therapy, mindfulness, cognitive training, and neurofeedback - for ADHD core symptoms and/or associated dysfunctions.[13]

Additionally, due to the paucity of RCTs, the NICE committee was not able to make any evidence-based recommendation on which type(s) of non-pharmacological treatments are preferred. This is highly problematic in particular for those patients who do not opt for or are unable to tolerate a pharmacologic treatment and need to be informed on the comparative efficacy/tolerability of currently available non-pharmacologic options. Furthermore, recent studies have assessed internet delivered non-pharmacological interventions, to possibly maximise efficiency and cost-effectiveness (e.g.,[14,15]). This is particularly relevant considering the need for remote assessment/treatment prompted by the current pandemic-related restrictions and the likely push towards digital interventions in the post COVID-19 era.

Therefore, there is a need for updated evidence synthesis regarding how non-pharmacological interventions - and different ways to deliver them - compare with each other, to pharmacologic treatments, or to combinations of pharmacological and non-pharmacological interventions in terms of efficacy and tolerability on specific relevant outcomes (e.g., ADHD core symptoms, emotional dysregulation, executive functions) in adults with ADHD.

A well-powered RCT would be suited to compare pharmacological and non-pharmacological options but there are obvious financial and logistic constraints in conducting well-powered RCTs comparing all interventions for ADHD in adults. Network meta-analysis (NMA), which allows for the comparison of two or more interventions even when they have not been compared head-to-head in the studies included in the meta-analysis,[16] provides a cost-effective, practical option to address this gap.

For peer review only

A scoping search in PubMed/Medline (PubMed Central and Europe PubMed Central), PsycInfo and Embase (up to October 1st, 2021) using search terms for "ADHD", "adults" and "network meta-analysis" did not find any NMA including, in the same network, pharmacologic and non-pharmacologic interventions for adults with ADHD. Based on our searches, no protocol for such NMA has been registered in PROSPERO or other registries at the time of writing.

Therefore, we aim to conduct the first systematic review/NMA of published and unpublished RCTs to assess the comparative efficacy and tolerability of UK licensed and unlicensed medications for ADHD, non-pharmacological treatments, or their combination, on ADHD core symptoms severity and related dysfunctions (e.g., emotional dysregulation) in adults with ADHD.

METHODS AND ANALYSIS

The methods of the proposed study are based on the Preferred Reporting Items for Systematic Reviews and Meta-Analyses (PRISMA) statement and its extension for Network Meta-Analyses.[17] The methods are in line with those of another NMA[18] of pharmacological and non-pharmacological interventions for depressive disorder.

The protocol of the present NMA is pre-registered in PROSPERO [ANONYMISED].[19]

Search

We will update the search for RCTs of medications for ADHD from an NMA[9] published in 2018 and conduct a *de novo* search for the non-pharmacological interventions. The search will be conducted with the support of Systematic Review Solutions Ltd. (SRS), an independent health research service company specialising in evidence-based medicine methods and meta-research training, production of systematic reviews and Health

For peer review only - http://bmjopen.bmj.com/site/about/guidelines.xhtml

Technology Assessment reports, and development of clinical practice guidelines. SRS conducted the search for the previous NMA.[9] Using a similar search strategy, we will search a broad set of electronic databases, including: PubMed, BIOSIS Previews, CINAHL, the Cochrane Central Register of Controlled Trials, EMBASE, ERIC, MEDLINE, PsycINFO, OpenGrey, Web of Science Core Collection, ProQuest Dissertations and Theses (UK and Ireland), ProQuest Dissertations and Theses, and the WHO International Trials Registry Platform, including *ClinicalTrials.gov*, with no language/type of document restrictions. We will use the following syntax in Pubmed, and adapt it for other databases: (Attention-Deficit/Hyperactivity Disorder OR Attention-Deficit OR Attention Deficit OR ADHD OR Hyperkinetic Syndrome OR Hyperkinetic Disorder) AND random* (there will not be any specific terms for the type of intervention – to allow any type of intervention tested in RCTs), with the age filter ADULT (19+ years).

We will also search the US Food and Drug Administration, European Medicines Agency, and relevant drug manufacturers' websites for RCTs of medications. We will also endeavour to gather relevant unpublished data for pharmacological and non-pharmacological interventions by contacting drug companies, study authors and the members of key scientific organisations of ADHD worldwide.

Selection criteria

Study design: We will include parallel or cross-over RCTs of at least 1 week duration for pharmacological treatment, in line with prior work,[9] and of at least 4 sessions for psychotherapy. For trials of neurotherapies (e.g., neurofeedback) we will include RCTs of any length deemed appropriate for these approaches. For trials of medications, cognitive training or neurotherapies alone, we will include only double-blind RCTs (patients and raters blinded). For trials of psychotherapy alone or the combination of medications and

For peer review only

psychotherapy, we will include trials in which observers and/or raters were masked and/or participants were assessed by self-rating ADHD scales, because participants and therapists cannot be blinded, but we will then conduct a sensitivity analysis including only double blind RCTs (please see below). We will exclude studies with enrichment designs (e.g., trials selecting responders only after a run-in phase), because these types of trial can potentially inflate efficacy and tolerability estimates.[20]

Participants: We will retain RCTs including adults (≥18 years) with a formal diagnosis of ADHD according to DSM-III, DSM III-R, DSM-IV(TR), DSM-5, ICD-9, ICD-10 or ICD-11. We will not restrict our search by ADHD subtype or presentation, sex, ethnicity, intelligence quotient, socioeconomic status, or comorbidities.

Interventions: As in prior work,[9] pharmacological interventions will include: stimulants (methylphenidate and amphetamines, including lisdexamphetamine); atomoxetine; guanfacine XR, clonidine, bupropion, and modafinil. We will also search for eligible studies of viloxazine, which has bene recently approved by the FDA for children and adolescents (aged 6 to 17) with ADHD.[21] In the analyses, we will lump methylphenidate and amphetamines as: 1) a previous NMA[9] did not find any significant difference, in terms of efficacy, between methylphenidate and amphetamines in adults with ADHD; 2) accordingly, current NICE guidelines[11] recommend methylphenidate or lisdexamphetamine (or other amphetamines) as first-line pharmacological treatment for adults. Any type of non-pharmacological intervention will be considered.

Controls: The pharmacological control condition will be a pill placebo; non-pharmacological controls will include waiting list, treatment as usual, clinical management, active control in psychotherapy, and psychological placebo (sham).

Outcomes: Primary outcomes: 1) change in severity of ADHD core symptoms, according to a standardised rating scale.[9] We will consider separately self-rated ADHD core symptoms and

For peer review only

observer as well as clinician rated symptoms; 2) acceptability (all-cause discontinuation measured by the proportion of patients who withdrew from the study for any reason). Secondary outcomes: tolerability (drop-out due to side effects); change in the severity of emotional dysregulation, measured with any of the scales listed in Lenzi et al. [10]; executive dysfunctions, based on any of the scales in Tamminga et al.,[22] and quality of life, measured with any of the scales listed in Tsujii et al.[23]

Data collection

We will select studies, and extract/collect data in a two-step process. First, two independent investigators will screen the titles and abstracts we identified. Second, two independent investigators will obtain and read the full texts of all potentially relevant studies and determine the final list of studies to include. Any disagreement will be resolved by senior investigators. We will extract data into pre-specified data extraction forms.[24] For each study, we will extract information on study characteristics (e.g., setting, study design, sample size), participant characteristics (e.g., mean age and range, presence of comorbidities, concomitant therapies), interventions and controls (e.g., dose, frequency of treatment), and outcomes. We will systematically contact study when needed to gather unpublished information/data.

Study risk of bias assessment

The risk of bias in each individual RCT included in our NMA will be assessed using the Cochrane Risk of Bias tool-version 2 (RoB-2), as recommended in The Cochrane Handbook of Systematic Reviews of Interventions.[25] The tool includes five domains through which bias might be introduced into the result. For individually randomized trials (including cross-over trials), these include:

(1) bias arising from the randomization process;

(2) bias due to deviations from intended interventions;

For peer review only

(3) bias due to missing outcome data;

(4) bias in measurement of the outcome;

(5) bias in selection of the reported result.

We will use the appropriate templates for randomized parallel-group and cross-over trials, respectively.

Measures of treatment effects

For continuous outcomes, we will use mean difference (MD) as a measurement of treatment effect, with the relative 95% confidence interval (CI), when studies assessed the outcome with the same instruments; standardised mean difference (SMD, Cohen's d) when studies used different instruments.[26] We will use published mean values and standard deviations (SDs). If SDs are not available, they will be estimated by conversion from standard errors (SEs), p values, CIs or t-values.[27] If none of the above values is available from the published paper, we will contact the authors of the study to obtain information. If the information is not provided by the study author, we will employ a validated method for imputation to derive missing SDs.[28]

For dichotomous outcomes, we will calculate the odds ratio (OR) and relative 95% CI. Missing dichotomous outcome data will be handled according to the intention-to-treat principle. Participants who drop out after randomization will be considered as having a negative outcome.

Assessment of clinical and methodological heterogeneity within treatment comparisons

In each pairwise comparison, patient characteristics, treatments and outcome definitions of included studies should be similar.[26] We will produce descriptive statistics for studies and

For peer review only

study population characteristics across included trials to assess clinical and methodological heterogeneity. Within each pairwise comparison, we will compare these characteristics to assess the presence of clinical heterogeneity.

Assessment of transitivity across treatment comparisons

It is appropriate to use NMA if the assumption of transitivity can be defended. Transitivity holds when the distributions of the potential effect modifiers, like study and patient-level covariates, are balanced across all pairwise comparisons.[29,30] To assess the transitivity assumption, we will compare the distribution of clinical and methodological variables (e.g., ADHD severity at baseline, comorbidities, adherence, and treatment duration) that could act as effect modifiers across treatment comparisons.

Data analysis

First, we will conduct conventional pairwise meta-analyses with a random-effects model in STATA V.16.1 for all outcomes and comparisons with at least two studies. Then, we will perform Bayesian network meta-analysis for each outcome with random-effects model in OpenBUGS,[31] accounting for correlations induced by multi-arm studies.[32] Pooled estimates of network meta-analysis will be obtained using the Markov Chains Monte Carlo method. We will employ the binomial (dichotomous outcomes) and normal (continuous outcomes) likelihood functions and will use vague prior distributions for the treatment effects and a minimally informative prior distribution for the common heterogeneity SD depending on the outcome. We will examine Gelman-Rubin trace plots to check that multiple chains achieve convergence. All results will be reported as treatment effects (MD, SMD or OR) and their 95% credible intervals (CrI). NMA results will be presented in league tables and forest plots.[33]

For peer review only

We will estimate heterogeneity variances for each pairwise comparison in standard pairwise meta-analyses and assess the presence of statistical heterogeneity by visually inspecting the forest plots and calculating the I-squared statistic.[34] In the NMA, we will assume a common estimate for heterogeneity variance across comparisons and base our assessment of statistical heterogeneity in the whole network by comparing the magnitude of the common heterogeneity variance (τ2) with the empirical distribution as derived by Rhodes and Turner.[35,36] We will also calculate the total I-squared statistic.

Statistical disagreement between direct and indirect effect sizes (incoherence) will be evaluated globally, by comparison of the fit and parsimony of consistency and inconsistency models, and locally, by calculation of the difference between direct and indirect estimates in all closed loops in the network.[37] The node splitting method, which separates evidence on a particular comparison into direct and indirect evidence, will be used to calculate the inconsistency of the model.[38] To determine whether the results are affected by possible effect modifiers, we will conduct network meta-regression and subgroup analysis according to the following variables: study sponsorship, treatment duration, comorbid psychiatric disorders, study risk of bias, mean baseline severity, and percentage of participants treated with stable doses of medications in non-pharmacological RCTs.

We will then use the Surface Under the Cumulative RAnking curve (SUCRA) to measure, for any outcome, the probability each treatment is the best option among all treatments included in the network treatment and express the SUCRA measure as a percentage.[39] We will use a comparison-adjusted funnel plot for active treatments versus control to determine the possibility of small study-effects.[33,40]

We will assess the certainty of evidence derived using CINeMA (http://cinema.ispm.ch/).[41,42] CINeMA is a software which uses the netmeta R-package for performing Network meta-analysis of the data. The tool considers the following domains: within-study bias, across-

For peer review only

studies bias, indirectness, imprecision, heterogeneity and incoherence. It classifies overall confidence in evidence for each comparison as high, moderate, low, or very low.

We will finally conduct sensitivity analyses for primary outcomes by excluding trials without unpublished data, trials with imputed data, trials with overall sample size smaller than 20, and trials with non-blinded assessments.

Patient and Public Involvement

Our PPI co-author, Ms. [ANONYMISED], CEO of [ANONYMISED], one of the largest UK charitable associations of patients with ADHD, has played a central role in the development of this protocol since its initial design. During the preparation of the present proposal, Ms. [ANONYMISED]: • liaised with representatives (patients) of [ANONYMISED], and ADHD Europe to gather their feedback on the proposal; • based on the feedback form patients, critically commented on the overarching plan of the application, highlighting that it covers an important gap perceived as crucial by patients with ADHD and their families; • noted the importance of comparing different types of non-pharmacological interventions given the patchy provision across the UK and uncertainties around the evidence base supporting at least some types of nonpharmacological approaches; • recommended inclusion of quality of life as a secondary outcome measure.

As this is a protocol, no patients were directly involved in this study.

ETHICS AND DISSEMINATION

Ethics

As this is the protocol of an aggregate-data level NMA, no ethical approval will be needed.

Dissemination

For peer review only

Upon publication, the full dataset of the NMA will be available online freely in Mendeley Data, a secure online repository for research data. The results of the study will be disseminated nationally, via conferences organised by groups of people with lived experience (e.g., National Attention Deficit Disorder Information and Support Service and ADHD Foundation) and professional organisations (e.g., UKAAN, Royal College of Psychiatrists). Results will also be disseminated internationally via conferences (for service users: e.g., annual meeting of ADHD Europe; for professionals, e.g.: meetings of the WFA, Eunethydis, APSARD) and publications in peer-reviewed journals in the field of psychiatry/psychology and general medicine.

For peer review only

REFERENCES

1. Polanczyk GV, Willcutt EG, Salum GA, Kieling C, Rohde LA. ADHD prevalence estimates across three decades: an updated systematic review and meta-regression analysis. *Int J Epidemiol.* 2014;43(2):434-442.
2. Thomas R, Sanders S, Doust J, Beller E, Glasziou P. Prevalence of attention-deficit/hyperactivity disorder: a systematic review and meta-analysis. *Pediatrics.* 2015;135(4):e994-1001.
3. Faraone SV, Biederman J, Mick E. The age-dependent decline of attention deficit hyperactivity disorder: a meta-analysis of follow-up studies. *Psychol Med.* 2006;36(2):159-165.
4. Sibley MH, Mitchell JT, Becker SP. Method of adult diagnosis influences estimated persistence of childhood ADHD: a systematic review of longitudinal studies. *Lancet Psychiatry.* 2016;3(12):1157-1165.
5. Song P, Zha M, Yang Q, Zhang Y, Li X, Rudan I. The prevalence of adult attention-deficit hyperactivity disorder: A global systematic review and meta-analysis. *J Glob Health.* 2021;11:04009.
6. Faraone SV, Asherson P, Banaschewski T, et al. Attention-deficit/hyperactivity disorder. *Nat Rev Dis Primers.* 2015;1:15020.
7. Daley D, Jacobsen RH, Lange AM, Sørensen A, Walldorf J. The economic burden of adult attention deficit hyperactivity disorder: A sibling comparison cost analysis. *Eur Psychiatry.* 2019;61:41-48.
8. Cortese S. Pharmacologic Treatment of Attention Deficit-Hyperactivity Disorder. *N Engl J Med.* 2020;383(11):1050-1056.
9. Cortese S, Adamo N, Del Giovane C, et al. Comparative efficacy and tolerability of medications for attention-deficit hyperactivity disorder in children, adolescents, and adults: a systematic review and network meta-analysis. *Lancet Psychiatry.* 2018;5(9):727-738.
10. Lenzi F, Cortese S, Harris J, Masi G. Pharmacotherapy of emotional dysregulation in adults with ADHD: A systematic review and meta-analysis. *Neurosci Biobehav Rev.* 2018;84:359-367.
11. (NICE) NIfHaCE. Attention deficit hyperactivity disorder: diagnosis and management. https://www.nice.org.uk/guidance/ng87. Published 2018. Accessed.
12. Philipsen A, Jans T, Graf E, et al. Effects of Group Psychotherapy, Individual Counseling, Methylphenidate, and Placebo in the Treatment of Adult Attention-Deficit/Hyperactivity Disorder: A Randomized Clinical Trial. *JAMA Psychiatry.* 2015;72(12):1199-1210.
13. Nimmo-Smith V, Merwood A, Hank D, et al. Non-pharmacological interventions for adult ADHD: a systematic review. *Psychol Med.* 2020;50(4):529-541.
14. Moell BK, L; Nasri, B; Lindefors, N; Kaldo, V. Living SMART — A randomized controlled trial of a guided online course teaching adults with ADHD or sub-clinical ADHD to use smartphones to structure their everyday life. *Internet Interventions.* 2015;2:24-31.
15. Pettersson R, Söderström S, Edlund-Söderström K, Nilsson KW. Internet-Based Cognitive Behavioral Therapy for Adults With ADHD in Outpatient Psychiatric Care. *J Atten Disord.* 2017;21(6):508-521.

For peer review only

16. Mavridis D, Giannatsi M, Cipriani A, Salanti G. A primer on network meta-analysis with emphasis on mental health. *Evid Based Ment Health.* 2015;18(2):40-46.
17. Hutton B, Salanti G, Caldwell DM, et al. The PRISMA extension statement for reporting of systematic reviews incorporating network meta-analyses of health care interventions: checklist and explanations. *Ann Intern Med.* 2015;162(11):777-784.
18. Zhou X, Teng T, Zhang Y, et al. Comparative efficacy and acceptability of antidepressants, psychotherapies, and their combination for acute treatment of children and adolescents with depressive disorder: a systematic review and network meta-analysis. *Lancet Psychiatry.* 2020;7(7):581-601.
19. https://www.crd.york.ac.uk/PROSPERO/display_record.php?RecordID=265576.
20. Wong ICK, Banaschewski T, Buitelaar J, et al. Emerging challenges in pharmacotherapy research on attention-deficit hyperactivity disorder-outcome measures beyond symptom control and clinical trials. *Lancet Psychiatry.* 2019;6(6):528-537.
21. Findling RL, Candler SA, Nasser AF, et al. Viloxazine in the Management of CNS Disorders: A Historical Overview and Current Status. *CNS Drugs.* 2021;35(6):643-653.
22. Tamminga HG, Reneman L, Huizenga HM, Geurts HM. Effects of methylphenidate on executive functioning in attention-deficit/hyperactivity disorder across the lifespan: a meta-regression analysis. *Psychol Med.* 2016;46(9):1791-1807.
23. Tsujii N, Okada T, Usami M, et al. Effect of Continuing and Discontinuing Medications on Quality of Life After Symptomatic Remission in Attention-Deficit/Hyperactivity Disorder: A Systematic Review and Meta-Analysis. *J Clin Psychiatry.* 2020;81(3).
24. Pedder H, Sarri G, Keeney E, Nunes V, Dias S. Data extraction for complex meta-analysis (DECiMAL) guide. *Syst Rev.* 2016;5(1):212.
25. Higgins JT, J; Chandler, J; Cumpston, M; Li, T; Page, MJ; Welch, VA. Cochrane Handbook for Systematic Reviews of Interventions version 6.2 (updated February 2021). Cochrane, 2021. Available from www.training.cochrane.org/handbook. Published 2021. Accessed.
26. Higgins JPT, Green S, (editors). *Cochrane Handbook for Systematic Reviews of Interventions Version 5.1.0 [updated March 2011]. The Cochrane Collaboration. 2011 Available from www.handbook.cochrane.org.*
27. Altman DG, Bland JM. Detecting skewness from summary information. *Bmj.* 1996;313(7066):1200.
28. Furukawa TA, Barbui C, Cipriani A, Brambilla P, Watanabe N. Imputing missing standard deviations in meta-analyses can provide accurate results. *J Clin Epidemiol.* 2006;59(1):7-10.
29. Cipriani A, Higgins JP, Geddes JR, Salanti G. Conceptual and technical challenges in network meta-analysis. *Ann Intern Med.* 2013;159(2):130-137.
30. Salanti G. Indirect and mixed-treatment comparison, network, or multiple-treatments meta-analysis: many names, many benefits, many concerns for the next generation evidence synthesis tool. *Res Synth Methods.* 2012;3(2):80-97.
31. Lunn D, Spiegelhalter D, Thomas A, Best N. The BUGS project: Evolution, critique and future directions. *Stat Med.* 2009;28(25):3049-3067.
32. Lumley T. Network meta-analysis for indirect treatment comparisons. *Stat Med.* 2002;21(16):2313-2324.
33. Chaimani A, Higgins JP, Mavridis D, Spyridonos P, Salanti G. Graphical tools for network meta-analysis in STATA. *PLoS One.* 2013;8(10):e76654.

For peer review only - http://bmjopen.bmj.com/site/about/guidelines.xhtml

34. Higgins JP, Thompson SG, Deeks JJ, Altman DG. Measuring inconsistency in meta-analyses. *BMJ.* 2003;327(7414):557-560.
35. Rhodes KM, Turner RM, Higgins JP. Predictive distributions were developed for the extent of heterogeneity in meta-analyses of continuous outcome data. *J Clin Epidemiol.* 2015;68(1):52-60.
36. Turner RM, Davey J, Clarke MJ, Thompson SG, Higgins JP. Predicting the extent of heterogeneity in meta-analysis, using empirical data from the Cochrane Database of Systematic Reviews. *Int J Epidemiol.* 2012;41(3):818-827.
37. Veroniki AA, Vasiliadis HS, Higgins JP, Salanti G. Evaluation of inconsistency in networks of interventions. *Int J Epidemiol.* 2013;42(1):332-345.
38. Higgins JP, Jackson D, Barrett JK, Lu G, Ades AE, White IR. Consistency and inconsistency in network meta-analysis: concepts and models for multi-arm studies. *Res Synth Methods.* 2012;3(2):98-110.
39. Salanti G, Ades AE, Ioannidis JPA. Graphical methods and numerical summaries for presenting results from multiple-treatment meta-analysis: an overview and tutorial. *J Clin Epidemiol.* 2011;64(2):163-171.
40. Peters JL, Sutton AJ, Jones DR, Abrams KR, Rushton L. Contour-enhanced meta-analysis funnel plots help distinguish publication bias from other causes of asymmetry. *J Clin Epidemiol.* 2008;61(10):991-996.
41. Salanti G, Del Giovane C, Chaimani A, Caldwell DM, Higgins JP. Evaluating the quality of evidence from a network meta-analysis. *PLoS One.* 2014;9(7):e99682.
42. Nikolakopoulou A, Higgins JPT, Papakonstantinou T, et al. CINeMA: An approach for assessing confidence in the results of a network meta-analysis. *PLoS Med.* 2020;17(4):e1003082.

For peer review only

Authors' contributions: SCo drafted the first version of the manuscript. The other co-authors critically revised it.

Funding statement: There is no currently funding for the project. SRC's role in this study was funded by a Wellcome Trust Clinical Fellowship (110049/Z/15/Z & 110049/Z/15/A). AC is supported by the National Institute for Health Research (NIHR) Oxford Cognitive Health Clinical Research Facility, by an NIHR Research Professorship (grant RP-2017-08-ST2-006), by the NIHR Oxford and Thames Valley Applied Research Collaboration and by the NIHR Oxford Health Biomedical Research Centre (grant BRC-1215-20005). The views expressed are those of the authors and not necessarily those of the UK National Health Service, the NIHR, or the UK Department of Health.

Competing interests statement: SCo declares honoraria and reimbursement for travel and accommodation expenses for lectures from the following non-profit associations: Association for Child and Adolescent Central Health (ACAMH), Canadian ADHD Alliance Resource (CADDRA), British Association of Pharmacology (BAP), and from Healthcare Convention for educational activity on ADHD. SRC receives honoraria from Elsevier for editorial work. He previously consulted for Promentis. SRC receives honoraria from Elsevier for editorial work. He previously consulted for Promentis. AC has received research and consultancy fees from INCiPiT (Italian Network for Paediatric Trials), CARIPLO Foundation and Angelini Pharma, outside the submitted work. The other authors declare no competing conflicts of interest.

For peer review only

Reporting checklist for protocol of a systematic review and meta analysis.

Based on the PRISMA-P guidelines.

Instructions to authors

Complete this checklist by entering the page numbers from your manuscript where readers will find each of the items listed below.

Your article may not currently address all the items on the checklist. Please modify your text to include the missing information. If you are certain that an item does not apply, please write "n/a" and provide a short explanation.

Upload your completed checklist as an extra file when you submit to a journal.

In your methods section, say that you used the PRISMA-Preporting guidelines, and cite them as:

Moher D, Shamseer L, Clarke M, Ghersi D, Liberati A, Petticrew M, Shekelle P, Stewart LA. Preferred Reporting Items for Systematic Review and Meta-Analysis Protocols (PRISMA-P) 2015 statement. Syst Rev. 2015;4(1):1.

		Reporting Item	Page Number
**Title**			
Identification	#1a	Identify the report as a protocol of a systematic review	1
Update	#1b	If the protocol is for an update of a previous systematic review, identify as such	N/A
**Registration**			
	#2	If registered, provide the name of the registry (such as PROSPERO) and registration number	3 and 7
**Authors**			
Contact	#3a	Provide name, institutional affiliation, e-mail address of all protocol authors; provide physical mailing address of corresponding author	1-2
Contribution	#3b	Describe contributions of protocol authors and identify the guarantor of the review	19

For peer review only

**Amendments**			
	#4	If the protocol represents an amendment of a previously completed or published protocol, identify as such and list changes; otherwise, state plan for documenting important protocol amendments	N/A
**Support**			
Sources	#5a	Indicate sources of financial or other support for the review	19
Sponsor	#5b	Provide name for the review funder and / or sponsor	19
Role of sponsor or funder	#5c	Describe roles of funder(s), sponsor(s), and / or institution(s), if any, in developing the protocol	N/A
**Introduction**			
Rationale	#6	Describe the rationale for the review in the context of what is already known	6-8
Objectives	#7	Provide an explicit statement of the question(s) the review will address with reference to participants, interventions, comparators, and outcomes (PICO)	8
**Methods**			
Eligibility criteria	#8	Specify the study characteristics (such as PICO, study design, setting, time frame) and report characteristics (such as years considered, language, publication status) to be used as criteria for eligibility for the review	9-10
Information sources	#9	Describe all intended information sources (such as electronic databases, contact with study authors, trial registers or other grey literature sources) with planned dates of coverage	8-9
Search strategy	#10	Present draft of search strategy to be used for at least one electronic database, including planned limits, such that it could be repeated	9
Study records - data management	#11a	Describe the mechanism(s) that will be used to manage records and data throughout the review	11

For peer review only

Study records - selection process	#11b	State the process that will be used for selecting studies (such as two independent reviewers) through each phase of the review (that is, screening, eligibility and inclusion in meta-analysis) 11
Study records - data collection process	#11c	Describe planned method of extracting data from reports (such as piloting forms, done independently, in duplicate), any processes for obtaining and confirming data from investigators 11
Data items	#12	List and define all variables for which data will be sought (such as PICO items, funding sources), any pre-planned data assumptions and simplifications 10-11
Outcomes and prioritization	#13	List and define all outcomes for which data will be sought, including prioritization of main and additional outcomes, with rationale 10-11
Risk of bias in individual studies	#14	Describe anticipated methods for assessing risk of bias of individual studies, including whether this will be done at the outcome or study level, or both; state how this information will be used in data synthesis 11-12
Data synthesis	#15a	Describe criteria under which study data will be quantitatively synthesized 13-15
Data synthesis	#15b	If data are appropriate for quantitative synthesis, describe planned summary measures, methods of handling data and methods of combining data from studies, including any planned exploration of consistency (such as I2, Kendall's τ) 13-15
Data synthesis	#15c	Describe any proposed additional analyses (such as sensitivity or subgroup analyses, meta-regression) 15
Data synthesis	#15d	If quantitative synthesis is not appropriate, describe the type of summary planned N/A
Meta-bias(es)	#16	Specify any planned assessment of meta-bias(es) (such as publication bias across studies, selective reporting within studies) 11-12
Confidence in cumulative evidence	#17	Describe how the strength of the body of evidence will be assessed (such as GRADE) 14-15

For peer review only

None The PRISMA-P elaboration and explanation paper is distributed under the terms of the Creative Commons Attribution License CC-BY. This checklist can be completed online using https://www.goodreports.org/, a tool made by the EQUATOR Network in collaboration with Penelope.ai

For peer review only

BMJ Open

Pharmacological and non-pharmacological interventions for adults with ADHD: protocol for a systematic review and network meta-analysis

Journal:	*BMJ Open*
Manuscript ID	bmjopen-2021-058102.R1
Article Type:	Protocol
Date Submitted by the Author:	06-Jan-2022
Complete List of Authors:	Cortese, Samuele; University of Southampton<br>Del Giovane, Cinzia; University of Bern<br>Chamberlain, Samuel; University of Southampton<br>Philipsen, Alexandra; Rheinische Friedrich-Wilhelms-Universitat Bonn, Department of Psychiatry and Psychotherapy<br>Young, Susan; University of Reykjavik,<br>Bilbow, Andrea; Attention Deficit Disorder Information and Support Service<br>Cipriani, Andrea; University of Oxford, Department of Psychiatry
**Primary Subject Heading**:	Mental health
Secondary Subject Heading:	Mental health
Keywords:	Adult psychiatry < PSYCHIATRY, Impulse control disorders < PSYCHIATRY, Clinical trials < THERAPEUTICS

SCHOLARONE™ Manuscripts

Pharmacological and non-pharmacological interventions for adults with ADHD: protocol for a systematic review and network meta-analysis

Samuele Cortese [1-5], Cinzia Del Giovane [6-7], Samuel R. Chamberlain [8-9], Alexandra Philipsen [10], Susan Young [11-12], Andre Bilbow [13-14], Andrea Cipriani [15-17]

[1] School of Psychology, Centre for Innovation in Mental Health (CIMH), Faculty of Environmental and Life Sciences, University of Southampton, Highfield Campus, UK. samuele.cortese@soton.ac.uk

[2]Clinical and Experimental Sciences (CNS and Psychiatry), Faculty of Medicine, University of Southampton, Southampton, UK.

[3]Solent NHS Trust, Southampton, UK.

[4]Hassenfeld Children's Hospital at NYU Langone, New York University Child Study Center, New York, USA.

[5]Division of Psychiatry and Applied Psychology, School of Medicine, University of Nottingham, Nottingham, UK.

[6] Institute of Primary Health Care (BIHAM), University of Bern, Bern, Switzerland. cinzia.delgiovane@biham.unibe.ch

[7]Population Health Laboratory, University of Fribourg, Fribourg, Switzerland

[8] Department of Psychiatry, Faculty of Medicine, University of Southampton, Southampton, UK. S.R.Chamberlain@soton.ac.uk

[9] Southern Health NHS Foundation Trust, Southampton, UK.

[10] Department of Psychiatry and Psychotherapy, University of Bonn, Bonn, Germany. Alexandra.Philipsen@ukbonn.de

[11] Department of Psychology, Reykjavik University, Reykjavik, Iceland. SuzyYoung@aol.com

[12] ADHD Foundation, Liverpool, UK

[13] Attention Deficit Disorder Information and Support Service (ADDISS), Edgware, UK. andrea@addiss.co.uk

[14] ADHD-Europe, Brussels, Belgium.

[15] Department of Psychiatry, University of Oxford, Oxford, UK.
andrea.cipriani@psych.ox.ac.uk

[16] Oxford Health NHS Foundation Trust, Warneford Hospital, Oxford, UK.

[17] Oxford Precision Psychiatry Lab, NIHR Oxford Health Biomedical Research Centre, Oxford, UK.

Word count (main text): 2869

Address correspondence to: Prof. Samuele Cortese, School of Psychology, Centre for Innovation in Mental Health (CIMH), Faculty of Environmental and Life Sciences, University of Southampton, Highfield Campus, Building 44, Southampton, SO17 1BJ, UK, Phone: +44 (0) 2380599645, E-mail: Samuele.cortese@gmail.com

ABSTRACT

Introduction: It is unclear how pharmacological and non-pharmacological interventions compare with each other in terms of efficacy and tolerability for core symptoms and additional problems in adults with Attention-Deficit/Hyperactivity Disorder (ADHD). We aim to conduct the first network meta-analysis (NMA) comparing pharmacological and non-pharmacological interventions (or their combinations) in adults with ADHD.

Methods and analysis: We will follow the Preferred Reporting Items for Systematic Reviews and Meta-Analyses (PRISMA) statement for NMAs. We will search a broad set of electronic databases/registries and contact drug companies and experts in the field to retrieve published and unpublished randomised controlled trials (RCTs) (parallel or cross-over) of medications (either licensed or unlicensed) and any non-pharmacological intervention in adults (≥18 years) with ADHD. Primary outcomes will be: 1) change in severity of ADHD core symptoms, and 2) acceptability (all-cause discontinuation). Secondary outcomes will include tolerability (drop-out due to side effects) and change in the severity of emotional dysregulation, executive dysfunctions, and quality of life. The risk of bias in each individual RCT included in the NMA will be assessed using the Cochrane Risk of Bias tool-version 2. We will evaluate the transitivity assumption comparing the distribution of possible effect modifiers across treatment comparisons. We will perform Bayesian network meta-analysis for each outcome with random-effects model in OpenBUGS. Pooled estimates of network meta-analysis will be obtained using the Markov Chains Monte Carlo method. We will judge the credibility in the evidence derived from the NMA using the CINeMA tool (which includes assessment of publication bias). We will conduct a series of sensitivity analyses to assess the robustness of the findings.

For peer review only

Ethics and dissemination: As this is the protocol for an aggregate-data level NMA, ethical approval will not be required. Results will be disseminated at national/international conferences and in peer-reviewed journals.

Registration: PROSPERO: CRD42021265576

Keywords: ADHD; Treatment; pharmacological; adults; network meta-analysis

For peer review only

STRENGTHS AND LIMITATIONS OF THIS STUDY

- The study will be conducted by a team with extensive expertise in the clinical assessment and treatment of ADHD, as well as in advanced Network Meta-Analysis (NMA) statistics
- The protocol was designed and the study will be carried out with the involvement of patients and member of the public in the review team.
- We will include both published and unpublished data, systematically gathered by drug manufacturers and study authors
- We will include, as outcomes, both ADHD core symptoms and related clinical problems, thus increasing the ecologic validity of the study
- The main limitation is that the proposed NMA will include aggregate-level data, rather than individual patient level data.

For peer review only

INTRODUCTION

Attention-Deficit/Hyperactivity Disorder (ADHD) is the most common neurodevelopmental condition in children,[1,2] and its impairing symptoms persist into adulthood in up to ~75% of childhood cases.[3,4] Adult ADHD has a prevalence estimated at ~2.5%[5] and is commonly comorbid with other disorders (e.g., depression or anxiety[6]) and with problems such as emotional dysregulation, which are often the main trigger for a referral to clinical services.[6] If untreated, adult ADHD is associated with substantial societal burden, including significantly increased risk of unemployment, substance abuse, criminal acts, accidents, and mortality.[6] The personal and societal costs of untreated ADHD in adults are estimated at around £20,000/person/yr.[7]

Both pharmacological and non-pharmacological (e.g., psychological) treatments are available for adults with ADHD.[8] Pharmacological and non-pharmacological interventions should be considered to be complementary, rather than mutually exclusive options. For instance, whilst stimulants are considered highly effective in decreasing the severity of adult ADHD core symptoms over the short-medium term (effect size, ES: ~ 0.8),[9] their efficacy in the treatment of emotional dysregulation is lower (ES: ~ 0.3-0.5),[10] suggesting the need for additional pharmacological or non-pharmacological options.

The current ADHD guidelines from the National Institute for Health and Care Excellence (NICE) recommend pharmacotherapy (stimulants followed by the selective noradrenaline reuptake inhibitor atomoxetine) as first-line treatment options for adult ADHD, with psychological therapies as second option.[11] However, the recommendation on the sequencing of pharmacological and non-pharmacological options was based on one randomised clinical trial (RCT) only,[12] comparing head-to-head pharmacotherapy and psychological treatment, retrieved from a literature search (up to 28 April 2017) that is now outdated. Since the NICE guidelines were published, a number of RCTs have been published pointing to significant

For peer review only

efficacy and good tolerability of a variety of non-pharmacological interventions - including cognitive behavioural therapy, dialectic behavioural therapy, mindfulness, cognitive training, and neurofeedback - for ADHD core symptoms and/or associated dysfunctions.[13]
Additionally, due to the paucity of RCTs, the NICE committee was not able to make any evidence-based recommendation on which type(s) of non-pharmacological treatments are preferred. This is highly problematic in particular for those patients who do not opt for or are unable to tolerate a pharmacologic treatment and need to be informed on the comparative efficacy/tolerability of currently available non-pharmacologic options. Furthermore, recent studies have assessed internet delivered non-pharmacological interventions, to possibly maximise efficiency and cost-effectiveness (e.g.,[14,15]). This is particularly relevant considering the need for remote assessment/treatment prompted by the current pandemic-related restrictions and the likely push towards digital interventions in the post COVID-19 era.

Therefore, there is a need for updated evidence synthesis regarding how non-pharmacological interventions - and different ways to deliver them - compare with each other, to pharmacologic treatments, or to combinations of pharmacological and non-pharmacological interventions in terms of efficacy and tolerability on specific relevant outcomes (e.g., ADHD core symptoms, emotional dysregulation, executive functions) in adults with ADHD.

A well-powered RCT would be suited to compare pharmacological and non-pharmacological options but there are obvious financial and logistic constraints in conducting well-powered RCTs comparing all interventions for ADHD in adults. Network meta-analysis (NMA), which allows for the comparison of two or more interventions even when they have not been compared head-to-head in the studies included in the meta-analysis,[16] provides a cost-effective, practical option to address this gap.

For peer review only

A scoping search in PubMed/Medline (PubMed Central and Europe PubMed Central), PsycInfo and Embase (up to October 1st, 2021) using search terms for “ADHD”, “adults” and “network meta-analysis” did not find any NMA including, in the same network, pharmacologic and non-pharmacologic interventions for adults with ADHD. Based on our searches, no protocol for such NMA has been registered in PROSPERO or other registries at the time of writing.

Therefore, we aim to conduct the first systematic review/NMA of published and unpublished RCTs to assess the comparative efficacy and tolerability of UK licensed and unlicensed medications for ADHD, non-pharmacological treatments, or their combination, on ADHD core symptoms severity and related dysfunctions (e.g., emotional dysregulation) in adults with ADHD.

METHODS AND ANALYSIS

The methods of the proposed study are based on the Preferred Reporting Items for Systematic Reviews and Meta-Analyses (PRISMA) statement and its extension for Network Meta-Analyses.[17] The methods are in line with those of another NMA[18] of pharmacological and non-pharmacological interventions for depressive disorder.

The protocol of the present NMA is pre-registered in PROSPERO (CRD42021265576).[19]

We plan to start the study on March 1st, 2022 and to complete it by March 1st, 2024.

Search

We will update the search for RCTs of medications for ADHD from an NMA[9] published in 2018 and conduct a *de novo* search for the non-pharmacological interventions. The search will be conducted with the support of Systematic Review Solutions Ltd. (SRS), an independent health research service company specialising in evidence-based medicine methods and meta-research training, production of systematic reviews and Health

For peer review only - http://bmjopen.bmj.com/site/about/guidelines.xhtml

Technology Assessment reports, and development of clinical practice guidelines. SRS conducted the search for the previous NMA.[9] Using a similar search strategy, we will search a broad set of electronic databases, including: PubMed, BIOSIS Previews, CINAHL, the Cochrane Central Register of Controlled Trials, EMBASE, ERIC, MEDLINE, PsycINFO, OpenGrey, Web of Science Core Collection, ProQuest Dissertations and Theses (UK and Ireland), ProQuest Dissertations and Theses, and the WHO International Trials Registry Platform, including *ClinicalTrials.gov*, with no language/type of document restrictions. For the specific syntax for each database, see Supplement 1.

We will also search the US Food and Drug Administration, European Medicines Agency, and relevant drug manufacturers' websites for RCTs of medications. We will also endeavour to gather relevant unpublished data for pharmacological and non-pharmacological interventions by contacting drug companies, study authors and the members of key scientific organisations of ADHD worldwide. Specifically, we will contact the European Network of Hyperkinetic disorder (Eunethydis), the World ADHD Federation, the he American Professional Society of ADHD and Related Disorders (APSARD) (APSARD), and the Canadian ADHD Resource Alliance (CADDRA) to advertise the study and query about the existence of any eligible unpublished study.

Selection criteria

Study design: We will include parallel or cross-over RCTs of at least 1 week duration for pharmacological treatment, in line with prior work,[9] and of at least 4 sessions for psychotherapy. For cross-over studies of medications, to address concerns around possible ‘carry over’ effects, we will use data from the pre cross-over phase, when reported in the paper. When data for the pre cross-over phase are not reported, we will contact study authors to gather them. If pre cross-over data are not reported and not available on request, we will

For peer review only

use data at the end point (after crossing over), only if there was a washout period (as reported in Cortese et al. [9]) between the two phases (pre cross-over and post cross-over) of the RCT. For trials of neurotherapies (e.g., neurofeedback) we will include RCTs of any length deemed appropriate for these approaches. For trials of medications, cognitive training or neurotherapies alone, we will include only double-blind RCTs (patients and raters blinded). For trials of psychotherapy alone or the combination of medications and psychotherapy, we will include trials in which observers and/or raters were masked and/or participants were assessed by self-rating ADHD scales, because participants and therapists cannot be blinded, but we will then conduct a sensitivity analysis including only double blind RCTs (please see below). We will exclude studies with enrichment designs (e.g., trials selecting responders only after a run-in phase), because these types of trial can potentially inflate efficacy and tolerability estimates.[20]

Participants: We will retain RCTs including adults (≥18 years) with a formal diagnosis of ADHD according to DSM-III, DSM III-R, DSM-IV(TR), DSM-5, ICD-9, ICD-10 or ICD-11. We will not restrict our search by ADHD subtype or presentation, sex, ethnicity, intelligence quotient, socioeconomic status, or comorbidities.

Interventions: As in prior work,[9] pharmacological interventions will include: stimulants (methylphenidate and amphetamines, including lisdexamphetamine); atomoxetine; guanfacine XR, clonidine, bupropion, and modafinil. We will also search for eligible studies of viloxazine, which has bene recently approved by the FDA for children and adolescents (aged 6 to 17) with ADHD.[21] In the analyses, we will lump methylphenidate and amphetamines as: 1) a previous NMA[9] did not find any significant difference, in terms of efficacy, between methylphenidate and amphetamines in adults with ADHD; 2) accordingly, current NICE guidelines[11] recommend methylphenidate <u>or</u> lisdexamphetamine (or other

For peer review only - http://bmjopen.bmj.com/site/about/guidelines.xhtml

amphetamines) as first-line pharmacological treatment for adults. Any type of non-pharmacological intervention will be considered.

Controls: The pharmacological control condition will be a pill placebo; non-pharmacological controls will include waiting list, treatment as usual, clinical management, active control in psychotherapy, and psychological placebo (sham).

Outcomes: Primary outcomes: 1) change in severity of ADHD core symptoms, according to a standardised rating scale.[9] We will consider separately self-rated ADHD core symptoms and observer as well as clinician rated symptoms; 2) acceptability (all-cause discontinuation measured by the proportion of patients who withdrew from the study for any reason). Secondary outcomes: tolerability (drop-out due to side effects); change in the severity of emotional dysregulation, measured with any of the scales listed in Lenzi et al. [10]; executive dysfunctions, based on any of the scales in Tamminga et al.,[22] and quality of life, measured with any of the scales listed in Tsujii et al.[23]

Data collection

We will select studies, and extract/collect data in a two-step process. First, two independent investigators will screen the titles and abstracts we identified. Second, two independent investigators will obtain and read the full texts of all potentially relevant studies and determine the final list of studies to include. Any disagreement will be resolved by senior investigators. We will extract data into pre-specified data extraction forms.[24] For each study, we will extract information on study characteristics (e.g., setting, study design, sample size), participant characteristics (e.g., mean age and range, presence of comorbidities, concomitant therapies), interventions and controls (e.g., dose, frequency of treatment), and outcomes. We will systematically contact study when needed to gather unpublished information/data.

For peer review only

Study risk of bias assessment

The risk of bias in each individual RCT included in our NMA will be assessed using the Cochrane Risk of Bias tool-version 2 (RoB-2), as recommended in The Cochrane Handbook of Systematic Reviews of Interventions.[25] The tool includes five domains through which bias might be introduced into the result. For individually randomized trials (including cross-over trials), these include:

(1) bias arising from the randomization process;

(2) bias due to deviations from intended interventions;

(3) bias due to missing outcome data;

(4) bias in measurement of the outcome;

(5) bias in selection of the reported result.

We will use the appropriate templates for randomized parallel-group and cross-over trials, respectively.

Measures of treatment effects

For continuous outcomes, we will use mean difference (MD) as a measurement of treatment effect, with the relative 95% confidence interval (CI), when studies assessed the outcome with the same instruments; standardised mean difference (SMD, Cohen's d) when studies used different instruments.[26] We will use published mean values and standard deviations (SDs). If SDs are not available, they will be estimated by conversion from standard errors (SEs), p values, CIs or t-values.[27] If none of the above values is available from the published paper, we will contact the authors of the study to obtain information. If the information is not provided by the study author, we will employ a validated method for imputation to derive missing SDs.[28]

For peer review only

For dichotomous outcomes, we will calculate the odds ratio (OR) and relative 95% CI. Missing dichotomous outcome data will be handled according to the intention-to-treat principle. Participants who drop out after randomization will be considered as having a negative outcome.

Assessment of clinical and methodological heterogeneity within treatment comparisons

In each pairwise comparison, patient characteristics, treatments and outcome definitions of included studies should be similar.[26] We will produce descriptive statistics for studies and study population characteristics across included trials to assess clinical and methodological heterogeneity. Within each pairwise comparison, we will compare these characteristics to assess the presence of clinical heterogeneity.

Assessment of transitivity across treatment comparisons

It is appropriate to use NMA if the assumption of transitivity can be defended. Transitivity holds when the distributions of the potential effect modifiers, like study and patient-level covariates, are balanced across all pairwise comparisons.[29,30] To assess the transitivity assumption, we will compare the distribution of clinical and methodological variables (e.g., ADHD severity at baseline, comorbidities, adherence, and treatment duration) that could act as effect modifiers across treatment comparisons.

Data analysis

First, we will conduct conventional pairwise meta-analyses with a random-effects model in STATA V.16.1 for all outcomes and comparisons with at least two studies. Then, we will perform Bayesian network meta-analysis for each outcome with random-effects model in OpenBUGS,[31] accounting for correlations induced by multi-arm studies.[32] Pooled estimates

For peer review only

of network meta-analysis will be obtained using the Markov Chains Monte Carlo method. We will employ the binomial (dichotomous outcomes) and normal (continuous outcomes) likelihood functions and will use vague prior distributions for the treatment effects and a minimally informative prior distribution for the common heterogeneity SD depending on the outcome. We will examine Gelman-Rubin trace plots to check that multiple chains achieve convergence. All results will be reported as treatment effects (MD, SMD or OR) and their 95% credible intervals (CrI). NMA results will be presented in league tables and forest plots.[33]

We will estimate heterogeneity variances for each pairwise comparison in standard pairwise meta-analyses and assess the presence of statistical heterogeneity by visually inspecting the forest plots and calculating the I-squared statistic.[34] In the NMA, we will assume a common estimate for heterogeneity variance across comparisons and base our assessment of statistical heterogeneity in the whole network by comparing the magnitude of the common heterogeneity variance ($\tau 2$) with the empirical distribution as derived by Rhodes and Turner.[35,36] We will also calculate the total I-squared statistic.

Statistical disagreement between direct and indirect effect sizes (incoherence) will be evaluated globally, by comparison of the fit and parsimony of consistency and inconsistency models, and locally, by calculation of the difference between direct and indirect estimates in all closed loops in the network.[37] The node splitting method, which separates evidence on a particular comparison into direct and indirect evidence, will be used to calculate the inconsistency of the model.[38] To determine whether the results are affected by possible effect modifiers, we will conduct network meta-regression and subgroup analysis according to the following variables: study sponsorship, treatment duration, comorbid psychiatric disorders, study risk of bias, mean baseline severity, and percentage of participants treated with stable doses of medications in non-pharmacological RCTs.

For peer review only

We will then use the Surface Under the Cumulative RAnking curve (SUCRA) to measure, for any outcome, the probability each treatment is the best option among all treatments included in the network treatment and express the SUCRA measure as a percentage.[39] We will use a comparison-adjusted funnel plot for active treatments versus control to determine the possibility of small study-effects.[33,40]

We will assess the certainty of evidence derived using CINeMA (http://cinema.ispm.ch/).[41,42] CINeMA is a software which uses the netmeta R-package for performing Network meta-analysis of the data. The tool considers the following domains: within-study bias, publication bias, indirectness, imprecision, heterogeneity and incoherence. It classifies overall confidence in evidence for each comparison as high, moderate, low, or very low. In particular, for publication bias we will use the new tool implemented within CINeMA, ROB-MEN, that allow to evaluate the impact of this bias on the results of network meta-analyses of interventions.[43]

We will finally conduct sensitivity analyses for primary outcomes by excluding trials without unpublished data, trials with imputed data, trials with overall sample size smaller than 20, and trials with non-blinded assessments. We will also conduct a sensitivity analysis combing guanfacine and clonidine in the same node.

Patient and Public Involvement

Our PPI co-author, Ms. Bilbow, CEO of The National Attention Deficit Disorder Information and Support Service (ADDISS), one of the largest UK charities of patients with ADHD, has played a central role in the development of this protocol since its initial design. During the preparation of the present proposal, Ms. Bilbow: • liaised with representatives (patients) of ADDISS, and ADHD Europe to gather their feedback on the proposal; • based on the feedback form patients, critically commented on the overarching plan of the application,

For peer review only

highlighting that it covers an important gap perceived as crucial by patients with ADHD and their families; • noted the importance of comparing different types of non-pharmacological interventions given the patchy provision across the UK and uncertainties around the evidence base supporting at least some types of nonpharmacological approaches; • recommended inclusion of quality of life as a secondary outcome measure.

As this is a protocol, no patients were directly involved in this study.

ETHICS AND DISSEMINATION

Ethics

As this is the protocol of an aggregate-data level NMA, no ethical approval will be needed.

Dissemination

Upon publication, the full dataset of the NMA and the codes for the analyses will be available online freely in Mendeley Data, a secure online repository for research data. The results of the study will be disseminated nationally, via conferences organised by groups of people with lived experience (e.g., National Attention Deficit Disorder Information and Support Service and ADHD Foundation) and professional organisations (e.g., UKAAN, Royal College of Psychiatrists). Results will also be disseminated internationally via conferences (for service users: e.g., annual meeting of ADHD Europe; for professionals, e.g.: meetings of the WFA, Eunethydis, APSARD) and publications in peer-reviewed journals in the field of psychiatry/psychology and general medicine.

For peer review only

REFERENCES

1. Cortese S, Solmi M, Arrondo G, et al. Association between mental disorders and somatic conditions: protocol for an umbrella review. *Evid Based Ment Health* 2020;23:135-139.
2. Barican JL, Yung D, Schwartz C, et al. Prevalence of childhood mental disorders in high-income countries: a systematic review and meta-analysis to inform policymaking. *Evid Based Ment Health* Published Online First: 19 July 2021. doi: 10.1136/ebmental-2021-300277.
3. Zhang L, Lagerberg T, Chen Q, et al. Prediction of treatment dosage and duration from free-text prescriptions: an application to ADHD medications in the Swedish prescribed drug register. *Evid Based Ment Health* 2021;24:146-152.
4. Sibley MH, Mitchell JT, Becker SP. Method of adult diagnosis influences estimated persistence of childhood ADHD: a systematic review of longitudinal studies. *Lancet Psychiatry.* 2016;3(12):1157-1165.
5. Song P, Zha M, Yang Q, Zhang Y, Li X, Rudan I. The prevalence of adult attention-deficit hyperactivity disorder: A global systematic review and meta-analysis. *J Glob Health.* 2021;11:04009.
6. Faraone SV, Asherson P, Banaschewski T, et al. Attention-deficit/hyperactivity disorder. *Nat Rev Dis Primers.* 2015;1:15020.
7. Daley D, Jacobsen RH, Lange AM, Sørensen A, Walldorf J. The economic burden of adult attention deficit hyperactivity disorder: A sibling comparison cost analysis. *Eur Psychiatry.* 2019;61:41-48.
8. Cortese S. Pharmacologic Treatment of Attention Deficit-Hyperactivity Disorder. *N Engl J Med.* 2020;383(11):1050-1056.
9. Cortese S, Adamo N, Del Giovane C, et al. Comparative efficacy and tolerability of medications for attention-deficit hyperactivity disorder in children, adolescents, and adults: a systematic review and network meta-analysis. *Lancet Psychiatry.* 2018;5(9):727-738.
10. Lenzi F, Cortese S, Harris J, Masi G. Pharmacotherapy of emotional dysregulation in adults with ADHD: A systematic review and meta-analysis. *Neurosci Biobehav Rev.* 2018;84:359-367.
11. (NICE) NIfHaCE. Attention deficit hyperactivity disorder: diagnosis and management. https://www.nice.org.uk/guidance/ng87. Published 2018. Accessed.
12. Philipsen A, Jans T, Graf E, et al. Effects of Group Psychotherapy, Individual Counseling, Methylphenidate, and Placebo in the Treatment of Adult Attention-Deficit/Hyperactivity Disorder: A Randomized Clinical Trial. *JAMA Psychiatry.* 2015;72(12):1199-1210.
13. Nimmo-Smith V, Merwood A, Hank D, et al. Non-pharmacological interventions for adult ADHD: a systematic review. *Psychol Med.* 2020;50(4):529-541.
14. Moell BK, L; Nasri, B; Lindefors, N; Kaldo, V. Living SMART — A randomized controlled trial of a guided online course teaching adults with ADHD or sub-clinical ADHD to use smartphones to structure their everyday life. *Internet Interventions.* 2015;2:24-31.
15. Pettersson R, Söderström S, Edlund-Söderström K, Nilsson KW. Internet-Based Cognitive Behavioral Therapy for Adults With ADHD in Outpatient Psychiatric Care. *J Atten Disord.* 2017;21(6):508-521.

For peer review only

16. Xiang Y, Cipriani A, Teng T, et alComparative efficacy and acceptability of psychotherapies for post-traumatic stress disorder in children and adolescents: a systematic review and network meta-analysis. *Evid Based Ment Health* 2021;24:153-160.
17. Hutton B, Salanti G, Caldwell DM, et al. The PRISMA extension statement for reporting of systematic reviews incorporating network meta-analyses of health care interventions: checklist and explanations. *Ann Intern Med.* 2015;162(11):777-784.
18. Zhou X, Teng T, Zhang Y, et al. Comparative efficacy and acceptability of antidepressants, psychotherapies, and their combination for acute treatment of children and adolescents with depressive disorder: a systematic review and network meta-analysis. *Lancet Psychiatry.* 2020;7(7):581-601.
19. https://www.crd.york.ac.uk/PROSPERO/display_record.php?RecordID=265576.
20. Wong ICK, Banaschewski T, Buitelaar J, et al. Emerging challenges in pharmacotherapy research on attention-deficit hyperactivity disorder-outcome measures beyond symptom control and clinical trials. *Lancet Psychiatry.* 2019;6(6):528-537.
21. Findling RL, Candler SA, Nasser AF, et al. Viloxazine in the Management of CNS Disorders: A Historical Overview and Current Status. *CNS Drugs.* 2021;35(6):643-653.
22. Tamminga HG, Reneman L, Huizenga HM, Geurts HM. Effects of methylphenidate on executive functioning in attention-deficit/hyperactivity disorder across the lifespan: a meta-regression analysis. *Psychol Med.* 2016;46(9):1791-1807.
23. Tsujii N, Okada T, Usami M, et al. Effect of Continuing and Discontinuing Medications on Quality of Life After Symptomatic Remission in Attention-Deficit/Hyperactivity Disorder: A Systematic Review and Meta-Analysis. *J Clin Psychiatry.* 2020;81(3).
24. Pedder H, Sarri G, Keeney E, Nunes V, Dias S. Data extraction for complex meta-analysis (DECiMAL) guide. *Syst Rev.* 2016;5(1):212.
25. Higgins JT, J; Chandler, J; Cumpston, M; Li, T; Page, MJ; Welch, VA. Cochrane Handbook for Systematic Reviews of Interventions version 6.2 (updated February 2021). Cochrane, 2021. Available from www.training.cochrane.org/handbook. Published 2021. Accessed.
26. Solmi M, Wade TD, Byrne S, et al. Comparative efficacy and acceptability of psychological interventions for the treatment of adult outpatients with anorexia nervosa: a systematic review and network meta-analysis. *Lancet Psychiatry*. 2021;8(3):215-224. doi: 10.1016/S2215-0366(20)30566-6.
27. Altman DG, Bland JM. Detecting skewness from summary information. *Bmj.* 1996;313(7066):1200.
28. Furukawa TA, Barbui C, Cipriani A, Brambilla P, Watanabe N. Imputing missing standard deviations in meta-analyses can provide accurate results. *J Clin Epidemiol.* 2006;59(1):7-10.
29. Cipriani A, Higgins JP, Geddes JR, Salanti G. Conceptual and technical challenges in network meta-analysis. *Ann Intern Med.* 2013;159(2):130-137.
30. Salanti G. Indirect and mixed-treatment comparison, network, or multiple-treatments meta-analysis: many names, many benefits, many concerns for the next generation evidence synthesis tool. *Res Synth Methods.* 2012;3(2):80-97.
31. Lunn D, Spiegelhalter D, Thomas A, Best N. The BUGS project: Evolution, critique and future directions. *Stat Med.* 2009;28(25):3049-3067.
32. Lumley T. Network meta-analysis for indirect treatment comparisons. *Stat Med.* 2002;21(16):2313-2324.

For peer review only

33. Chaimani A, Higgins JP, Mavridis D, Spyridonos P, Salanti G. Graphical tools for network meta-analysis in STATA. *PLoS One.* 2013;8(10):e76654.
34. Higgins JP, Thompson SG, Deeks JJ, Altman DG. Measuring inconsistency in meta-analyses. *BMJ.* 2003;327(7414):557-560.
35. Rhodes KM, Turner RM, Higgins JP. Predictive distributions were developed for the extent of heterogeneity in meta-analyses of continuous outcome data. *J Clin Epidemiol.* 2015;68(1):52-60.
36. Turner RM, Davey J, Clarke MJ, Thompson SG, Higgins JP. Predicting the extent of heterogeneity in meta-analysis, using empirical data from the Cochrane Database of Systematic Reviews. *Int J Epidemiol.* 2012;41(3):818-827.
37. Veroniki AA, Vasiliadis HS, Higgins JP, Salanti G. Evaluation of inconsistency in networks of interventions. *Int J Epidemiol.* 2013;42(1):332-345.
38. Higgins JP, Jackson D, Barrett JK, Lu G, Ades AE, White IR. Consistency and inconsistency in network meta-analysis: concepts and models for multi-arm studies. *Res Synth Methods.* 2012;3(2):98-110.
39. Salanti G, Ades AE, Ioannidis JPA. Graphical methods and numerical summaries for presenting results from multiple-treatment meta-analysis: an overview and tutorial. *J Clin Epidemiol.* 2011;64(2):163-171.
40. Peters JL, Sutton AJ, Jones DR, Abrams KR, Rushton L. Contour-enhanced meta-analysis funnel plots help distinguish publication bias from other causes of asymmetry. *J Clin Epidemiol.* 2008;61(10):991-996.
41. Salanti G, Del Giovane C, Chaimani A, Caldwell DM, Higgins JP. Evaluating the quality of evidence from a network meta-analysis. *PLoS One.* 2014;9(7):e99682.
42. Nikolakopoulou A, Higgins JPT, Papakonstantinou T, et al. CINeMA: An approach for assessing confidence in the results of a network meta-analysis. *PLoS Med.* 2020;17(4):e1003082.
43. Chiocchia V, Nikolakopoulou A, Higgins JPT, et al. ROB-MEN: a tool to assess risk of bias due to missing evidence in network meta-analysis. *BMC Med.* 2021;19(1):304.

For peer review only

Authors' contributions: SCo and AC designed the study. SCh contributed to the design, in particular in relation to the pharmacological interventions. AP and SY contributed to the design, in particular in relation to the non- pharmacological interventions. CDG contributed to the design, in particular in relation to the statistical analyses. AB contributed to the design, in particular in relation to PPI input. SCo drafted the first version of the manuscript. SCh, AP, SY, CDG, AB and AC critically revised the first version. Conduct, acquisition of data, analysis, and interpretation of data are not applicable here as this is a protocol of a meta-analysis.

Funding statement: There is no currently funding for the project. SRC's role in this study was funded by a Wellcome Trust Clinical Fellowship (110049/Z/15/Z & 110049/Z/15/A). AC is supported by the National Institute for Health Research (NIHR) Oxford Cognitive Health Clinical Research Facility, by an NIHR Research Professorship (grant RP-2017-08-ST2-006), by the NIHR Oxford and Thames Valley Applied Research Collaboration and by the NIHR Oxford Health Biomedical Research Centre (grant BRC-1215-20005). The views expressed are those of the authors and not necessarily those of the UK National Health Service, the NIHR, or the UK Department of Health.

For peer review only

Competing interests statement: SCo declares honoraria and reimbursement for travel and accommodation expenses for lectures from the following non-profit associations: Association for Child and Adolescent Central Health (ACAMH), Canadian ADHD Alliance Resource (CADDRA), British Association of Pharmacology (BAP), and from Healthcare Convention for educational activity on ADHD. SRC receives honoraria from Elsevier for editorial work. He previously consulted for Promentis. SRC receives honoraria from Elsevier for editorial

work. He previously consulted for Promentis. AC has received research and consultancy fees from INCiPiT (Italian Network for Paediatric Trials), CARIPLO Foundation and Angelini Pharma, outside the submitted work. The other authors declare no competing conflicts of interest.

For peer review only

Search syntax for each database (in alphabetical order):

A. BIOSIS Previews
TOPIC: (adhd OR hkd OR addh OR hyperkine* OR "attention deficit*" OR hyper-activ* OR hyperactiv* OR overactive OR inattentive OR impulsiv*) AND **TOPIC:** AND **TOPIC:** (RCT OR ((clinical OR control*) NEAR/10 trial*) OR crossover OR "cross over" OR cross-over OR randomi* OR (random* NEAR/1 (allocat* OR assign* OR select*)) OR blind* OR placebo OR "control group")
Indexes=BIOSIS Previews Timespan=All years

B. EMBASE
1. exp Attention Deficit Disorder with Hyperactivity/ or (adhd or hkd or addh or hyperkine* or "attention deficit*" or hyper-activ* or hyperactiv* or overactive or inattentive or impulsiv*).ti,ab.
2. (random$ or factorial$ or crossover$ or (cross over$) or cross-over$ or placebo$ or (doubl$ adj blind$) or (singl$ adj blind$) or assign$ or allocat$ or volunteer$).mp. or crossover-procedure/ or double-blind procedure/ or randomized controlled trial/ or single-blind procedure/
3. limit 2 to human
4. 1 and 2 and 3
No limitations

C. ERIC
((SU.EXACT.EXPLODE("Attention Deficit Disorders") OR ti(adhd OR hkd OR addh OR hyperkine* OR "attention deficit*" OR hyper-activ* OR hyperactiv* OR overactive OR inattentive OR impulsiv*) OR ab(adhd OR hkd OR addh OR hyperkine* OR "attention deficit*" OR hyper-activ* OR hyperactiv* OR overactive OR inattentive OR impulsiv*)) AND (ti(RCT OR ((clinical OR control*) NEAR/10 trial*) OR crossover OR "cross over" OR cross-over OR randomi* OR (random* NEAR/1 (allocat* OR assign* OR select*)) OR blind* OR placebo OR "control group") OR ab(RCT OR ((clinical OR control*) NEAR/10 trial*) OR crossover OR "cross over" OR cross-over OR randomi* OR (random* NEAR/1 (allocat* OR assign* OR select*)) OR blind* OR placebo OR "control group"))
No limitations

D. International Clinical Trials Registry Platform (WHO ICTRP)
(adhd OR hkd OR addh OR hyperkine* OR "attention deficit*" OR hyper-activ* OR hyperactiv* OR overactive OR inattentive OR impulsiv*) in Condition Field AND

E. MEDLINE
1. exp Attention Deficit Disorder with Hyperactivity/ or (adhd or hkd or addh or hyperkine* or "attention deficit*" or hyper-activ* or hyperactiv* or overactive or inattentive or impulsiv*).ti,ab.
2. (randomized controlled trial or controlled clinical trial).pt. or random$.ab. or placebo.ab. or drug therapy.fs. or trial.ab. or groups.ab.
3. exp animals/ not humans.sh.
4. 2 not 3
5. 1 and 2 and 4
No limitations

F. ProQuest Dissertations & Theses: UK & Ireland and ProQuest Dissertations & Theses A&I
((ti(adhd OR hkd OR addh OR hyperkine* OR "attention deficit*" OR hyper-activ* OR hyperactiv* OR overactive OR inattentive OR impulsiv*) OR ab(adhd OR hkd OR addh OR hyperkine* OR "attention deficit*" OR hyper-activ* OR hyperactiv* OR overactive OR inattentive OR impulsiv*)) AND (ti(RCT OR ((clinical OR control*) NEAR/10 trial*) OR crossover OR "cross over" OR cross-over OR randomi* OR (random* NEAR/1 (allocat* OR assign* OR select*)) OR blind* OR placebo OR "control group") OR ab(RCT OR ((clinical OR control*) NEAR/10 trial*) OR crossover OR "cross over" OR cross-over OR randomi* OR (random* NEAR/1 (allocat* OR assign* OR select*)) OR blind* OR placebo OR "control group"))
No limitations

G. PsycINFO
1. exp Attention Deficit Disorder with Hyperactivity/ or (adhd or hkd or addh or hyperkine* or "attention deficit*" or hyper-activ* or hyperactiv* or overactive or inattentive or impulsiv*).ti,ab.

For peer review only

2. (double-blind or random* assigned or control).tw.
3. and/1-2
4. limit 3 to human
No limitations

H. PubMed
("Attention Deficit Disorder with Hyperactivity"[Mesh] OR adhd[tiab] OR hkd[tiab] OR addh[tiab] OR hyperkine*[tiab] OR "attention deficit*"[tiab] OR hyper-activ*[tiab] OR hyperactiv*[tiab] OR overactive[tiab] OR inattentive[tiab] OR impulsiv*[tiab]) AND (randomized controlled trial[pt] OR controlled clinical trial[pt] OR randomized[tiab] OR placebo[tiab] OR clinical trials as topic[mesh:noexp] OR randomly[tiab] OR trial[ti]) NOT (animals[mh] NOT humans[mh])
Filter : adult (19+)

I. SIGLE
(adhd OR hkd OR addh OR hyperkine* OR "attention deficit*" OR hyper-activ* OR hyperactiv* OR overactive OR inattentive OR impulsiv*)

J. Cochrane Library
#1 MeSH descriptor: [Attention Deficit Disorder with Hyperactivity] explode all trees
#2 (adhd or hkd or addh or hyperkine* or "attention deficit*" or hyper-activ* or hyperactiv* or overactive or inattentive or impulsiv*):ti,ab
No limitations

K. Web of Science
TOPIC: (adhd OR hkd OR addh OR hyperkine* OR "attention deficit*" OR hyper-activ* OR hyperactiv* OR overactive OR inattentive OR impulsiv*) AND **TOPIC:** (RCT OR ((clinical OR control*) NEAR/10 trial*) OR crossover OR "cross over" OR cross-over OR randomi* OR (random* NEAR/1 (allocat* OR assign* OR select*)) OR blind* OR placebo OR "control group")
Indexes=SCI-EXPANDED, SSCI, CPCI-S, CPCI-SSH Timespan=All years

For peer review only

Reporting checklist for protocol of a systematic review and meta analysis.

Based on the PRISMA-P guidelines.

Instructions to authors

Complete this checklist by entering the page numbers from your manuscript where readers will find each of the items listed below.

Your article may not currently address all the items on the checklist. Please modify your text to include the missing information. If you are certain that an item does not apply, please write "n/a" and provide a short explanation.

Upload your completed checklist as an extra file when you submit to a journal.

In your methods section, say that you used the PRISMA-Preporting guidelines, and cite them as:

Moher D, Shamseer L, Clarke M, Ghersi D, Liberati A, Petticrew M, Shekelle P, Stewart LA. Preferred Reporting Items for Systematic Review and Meta-Analysis Protocols (PRISMA-P) 2015 statement. Syst Rev. 2015;4(1):1.

		Reporting Item	Page Number
**Title**			
Identification	#1a	Identify the report as a protocol of a systematic review	1
Update	#1b	If the protocol is for an update of a previous systematic review, identify as such	N/A
**Registration**			
	#2	If registered, provide the name of the registry (such as PROSPERO) and registration number	3 and 7
**Authors**			
Contact	#3a	Provide name, institutional affiliation, e-mail address of all protocol authors; provide physical mailing address of corresponding author	1-2
Contribution	#3b	Describe contributions of protocol authors and identify the guarantor of the review	19

For peer review only

**Amendments**			
	#4	If the protocol represents an amendment of a previously completed or published protocol, identify as such and list changes; otherwise, state plan for documenting important protocol amendments	N/A
**Support**			
Sources	#5a	Indicate sources of financial or other support for the review	19
Sponsor	#5b	Provide name for the review funder and / or sponsor	19
Role of sponsor or funder	#5c	Describe roles of funder(s), sponsor(s), and / or institution(s), if any, in developing the protocol	N/A
**Introduction**			
Rationale	#6	Describe the rationale for the review in the context of what is already known	6-8
Objectives	#7	Provide an explicit statement of the question(s) the review will address with reference to participants, interventions, comparators, and outcomes (PICO)	8
**Methods**			
Eligibility criteria	#8	Specify the study characteristics (such as PICO, study design, setting, time frame) and report characteristics (such as years considered, language, publication status) to be used as criteria for eligibility for the review	9-10
Information sources	#9	Describe all intended information sources (such as electronic databases, contact with study authors, trial registers or other grey literature sources) with planned dates of coverage	8-9
Search strategy	#10	Present draft of search strategy to be used for at least one electronic database, including planned limits, such that it could be repeated	9
Study records - data management	#11a	Describe the mechanism(s) that will be used to manage records and data throughout the review	11

For peer review only

Study records - selection process	#11b	State the process that will be used for selecting studies (such as two independent reviewers) through each phase of the review (that is, screening, eligibility and inclusion in meta-analysis) 11
Study records - data collection process	#11c	Describe planned method of extracting data from reports (such as piloting forms, done independently, in duplicate), any processes for obtaining and confirming data from investigators 11
Data items	#12	List and define all variables for which data will be sought (such as PICO items, funding sources), any pre-planned data assumptions and simplifications 10-11
Outcomes and prioritization	#13	List and define all outcomes for which data will be sought, including prioritization of main and additional outcomes, with rationale 10-11
Risk of bias in individual studies	#14	Describe anticipated methods for assessing risk of bias of individual studies, including whether this will be done at the outcome or study level, or both; state how this information will be used in data synthesis 11-12
Data synthesis	#15a	Describe criteria under which study data will be quantitatively synthesized 13-15
Data synthesis	#15b	If data are appropriate for quantitative synthesis, describe planned summary measures, methods of handling data and methods of combining data from studies, including any planned exploration of consistency (such as I2, Kendall's τ) 13-15
Data synthesis	#15c	Describe any proposed additional analyses (such as sensitivity or subgroup analyses, meta-regression) 15
Data synthesis	#15d	If quantitative synthesis is not appropriate, describe the type of summary planned N/A
Meta-bias(es)	#16	Specify any planned assessment of meta-bias(es) (such as publication bias across studies, selective reporting within studies) 11-12
Confidence in cumulative evidence	#17	Describe how the strength of the body of evidence will be assessed (such as GRADE) 14-15

For peer review only

None The PRISMA-P elaboration and explanation paper is distributed under the terms of the Creative Commons Attribution License CC-BY. This checklist can be completed online using https://www.goodreports.org/, a tool made by the EQUATOR Network in collaboration with Penelope.ai

For peer review only
