# Supplementary material for: Pharmacological and non-pharmacological interventions for adults with ADHD: protocol for a systematic review and network meta-analysis
Source: BMJ Open. 2022 Mar 11;12(3):e058102. doi: 10.1136/bmjopen-2021-058102 (PMC8919448; doi:10.1136/bmjopen-2021-058102)
Supplement: Reviewer comments [file bmjopen-2021-058102.reviewer_comments.pdf]

PEER REVIEW HISTORY

BMJ Open publishes all reviews undertaken for accepted manuscripts. Reviewers are asked to complete a checklist review form (**http://bmjopen.bmj.com/site/about/resources/checklist.pdf**) and are provided with free text boxes to elaborate on their assessment. These free text comments are reproduced below.

ARTICLE DETAILS

**TITLE (PROVISIONAL)**	Pharmacological and non-pharmacological interventions for adults with ADHD: protocol for a systematic review and network meta-analysis
**AUTHORS**	Cortese, Samuele; Del Giovane, Cinzia; Chamberlain, Samuel; Philipsen, Alexandra; Young, Susan; Bilbow, Andrea; Cipriani, Andrea

VERSION 1 – REVIEW

**REVIEWER**	Astri Lundervold<br>University of Bergen, Department of Biological and Medical Psychology
**REVIEW RETURNED**	01-Nov-2021

**GENERAL COMMENTS**	The topic of the paper focuses on an important challenge in the ADHD research field: the lack of knowledge related to the efficacy and tolerability of pharmacological compared to non-pharmacological treatment for adults with ADHD. In this paper, the authors describe plans for a network meta-analysis (MMA), putting a critical light on NICE recommendations of using psychological therapies as second therapy option for adults with ADHD.<br><br>The rationale for the study is well described in the introduction. A scoping search is included to confirm that this paper presents a plan for the first systematic review/NMA of published and unpublished RCTs comparing the efficacy and tolerability of the two treatment approaches or a combination of the two on core ADHD symptoms and related dysfunctions in adults with ADHD. The paper reports selection criteria for the study design, participants, interventions, control conditions and outcome measures. The criteria are well described and open up for inclusion of a wide range of different non-pharmacological treatment efforts. Primary outcome measures related to symptoms and acceptability is extended by secondary outcomes like tolerability (drop-out due to side effects), emotional regulation, executive function and quality of life, referring to specific lists of measurements used to assess these outcomes. In the data collection phase, data will be extracted according to the DECiMAL guide, and the risk of bias within each of the RCTs according to the Cochrane Risk of Bias tool-version 2 (RoB-2). A check on the members of the author team shows that they should have the skills and expertise to handle the different parts of this study.<br><br>I have only a few questions to the authors.<br><br>1. In the presented syntax, the age-filter is set to 19+ years, while the age is set to ≥18 years when describing the participants. I

	assume that there are several relevant studies with 18 years as the cut-off. Would those studies be excluded?<br>2. How would the authors act to be sure that they have included unpublished results from less known research groups?<br>3. What are the plans for taking cultural differences into account?<br>4. I have to admit that a detailed evaluation of the data-analytic part of the study is beyond my expertise. But I assume that the code for the analytic part of the study will be openly accessible when the review is published?

**REVIEWER**	Xavier Castells<br>Universitat de Girona, Translab Research Group, Unitat de Farmacologia Clínica. Departament de Ciències Mèdiques. Facultat de Medicina
**REVIEW RETURNED**	19-Nov-2021

**GENERAL COMMENTS**	This article reports the protocol for a systematic review and network meta-analysis investigating the efficacy and safety of pharmacological and non-pharmacological interventions for adults with ADHD. This is a protocol of an ambitious and clinically relevant study and I only have minor comments that hopefully may improve the reporting quality of this article:<br>1. It is not clear how the results from crossover studies will be handled. Will the authors extract and analyze the results from the first study phase or end-of-study results?<br>2. I guess that the risk of bias will be grater in RCTs investigating psychotherapies than pharmacotherapies because the formers will be, by nature, open-label while most RCTs investigating pharmacotherapies will have a double-blind design. Furthermore, almost all cross-over studies will investigate pharmacotherapies. These imbalances may bias the results of the NMA.<br>3. To aggregate studies investigating methylphenidate and amphetamine derivatives is a wise decision as these drugs have similar mechanism of action and clinical effects, at least in adults (in children this decision would be arguable) . Nevertheless, it can be criticized as it will be difficult to derive clinical recommendations from the results of this group. What should clinicians prescribe if this groups is found to be the most efficacious and safe? Furthermore, why clonidine and guanfacine are not also aggregated? This would be meaningful as, unlike for methylphenidate and amphetamines, the number of RCTs investigating alpha-2 agonists is low, therefore combining them would increase the statistical power.<br>4. Publication bias can jeopardize the validity of any systematic review. Is it possible to investigate such bias in the context of a NMA? If so, including such analysis would improve the quality of the review.

VERSION 1 – AUTHOR RESPONSE

<u>Reply to Reviewer # 1:</u>

In the presented syntax, the age-filter is set to 19+ years, while the age is set to ≥18 years when describing the participants. I assume that there are several relevant studies with 18 years as the cut-off. Would those studies be excluded?

Re: We thank the reviewer for highlighting this issue. We would like to point out that the filter 19+ will be applied for Pubmed only (in this database, “adulthood” is defined as 19+). We will still be able to capture any relevant study where the cut-off will be 18 years via:

- the other databases
- for RCTs of pharmacological interventions, screening the list of included and excluded studies form our previous NMA (Cortese et al, Lancet Psychiatry, 2018)
- via a search in PubMed from April 7th, 2017 (date of the last search in Cortese et al., Lancet Psychiatry, 2018) without age limits

How would the authors act to be sure that they have included unpublished results from less known research groups?
Re: We have a strong track record in identifying and collecting unpublished data from research groups, scientific societies, study authors and pharmaceutical companies. We will follow our usual procedure and will also contact the most relevant scientific associations of ADHD to advertise the study and query about the existence of any eligible unpublished study. We have existing links with the following associations, which represent the main scientific organisations on ADHD worldwide: the European Network of Hyperkinetic disorder (Eunethydis), the World ADHD Federation,
the he American Professional Society of ADHD and Related Disorders (APSARD) (APSARD), and the Canadian ADHD Resource Alliance (CADDRA). We have added the following to the revised version of the manuscript (under the subsection "Search"): "Specifically, we will contact the European Network of Hyperkinetic disorder (Eunethydis), the World ADHD Federation,
the American Professional Society of ADHD and Related Disorders (APSARD) (APSARD), and the Canadian ADHD Resource Alliance (CADDRA) to advertise the study and query about the existence of any eligible unpublished study."

What are the plans for taking cultural differences into account?
Re: As mentioned in the section "participants", we will not restrict our search by ethnicity or socioeconomic status.

I have to admit that a detailed evaluation of the data-analytic part of the study is beyond my expertise. But I assume that the code for the analytic part of the study will be openly accessible when the review is published?
Re: The Reviewer is correct. We have clarified this in the "Dissemination" section of the revised manuscript.

<u>Reply to Reviewer # 2</u>
It is not clear how the results from crossover studies will be handled. Will the authors extract and analyze the results from the first study phase or end-of-study results?
Re: We thank the Reviewer for this important point. We have added that, in line with the approach we

followed in our previous NMA of ADHD medications (Cortese et al., Lancet Psychiatry 2018), "For cross-over studies of medications, to address concerns around possible 'carry over' effects, we will use data from the pre cross-over phase, when reported in the paper. When data for the pre cross-over phase are not reported, we will contact study authors to gather them. If pre cross-over data are not reported and not available on request, we will use data at the end point (after crossing over), only if there was a washout period (as reported in Cortese et al., [9]) between the two phases (pre cross-over and post cross-over) of the RCT"

I guess that the risk of bias will be grater in RCTs investigating psychotherapies than pharmacotherapies because the formers will be, by nature, open-label while most RCTs investigating pharmacotherapies will have a double-blind design. Furthermore, almost all cross-over studies will investigate pharmacotherapies. These imbalances may bias the results of the NMA.

Re: The reviewer is correct. This is an important issue that should be carefully addressed. We have already carried out network meta-analyses comparing pharmacological and non-pharmacological treatments in psychiatry (e.g., Cuijpers P, Oud M, Karyotaki E, Noma H, Quero S, Cipriani A, Arroll B, Furukawa TA. Psychologic Treatment of Depression Compared With Pharmacotherapy and Combined Treatment in Primary Care: A Network Meta-Analysis. Ann Fam Med. 2021 May-Jun;19(3):262-270. doi: 10.1370/afm.2676. PMID: 34180847; PMCID: PMC8118476) and we have already used Risk of Bias to assess the methodological robustness of our findings. We will highlight this also in the discussion section of our NMA. As reported in a methodological piece by our group (Del Giovane, Cortese, Cipriani, JAMA Psychiatry, 2019) performing a sensitivity analysis to assess the association of high RoB for lack of masking with the treatment effects may temper these concerns.

To aggregate studies investigating methylphenidate and amphetamine derivatives is a wise decision as these drugs have similar mechanism of action and clinical effects, at least in adults (in children this decision would be arguable) . Nevertheless, it can be criticized as it will be difficult to derive clinical recommendations from the results of this group. What should clinicians prescribe if this groups is found to be the most efficacious and safe? Furthermore, why clonidine and guanfacine are not also aggregated? This would be meaningful as, unlike for methylphenidate and amphetamines, the number of RCTs investigating alpha-2 agonists is low, therefore combining them would increase the statistical power.

Re: We thank the Reviewer for this important remark. We are glad to hear that the Reviewer deems that combining methylphenidate and amphetamine derivatives is a wise decision and indeed based on a scooping search and preliminary geometry estimation of the network, it is not possible to do otherwise. Importantly, our previous NMA of ADHD medications, Cortese et al. 2018 (and its future update) has addressed /will address the comparative efficacy and tolerability of MPH vs amphetamines derivatives. We thank the Reviewer for the suggestion regarding clonidine and

guanfacine. We have added that we plan a sensitivity analysis combining these two compounds, as follows: “We will also conduct a sensitivity analysis combing guanfacine and clonidine in the same node.”

Publication bias can jeopardize the validity of any systematic review. Is it possible to investigate such bias in the context of a NMA? If so, including such analysis would improve the quality of the review.

Re: we agree with the reviewer and we have clarified that the assessment of publication bias is included in the CINeMA tool.

VERSION 2 – REVIEW

**REVIEWER**	Xavier Castells<br>Universitat de Girona, Translab Research Group, Unitat de Farmacologia Clínica. Departament de Ciències Mèdiques. Facultat de Medicina
**REVIEW RETURNED**	25-Jan-2022

**GENERAL COMMENTS**	The authors have addressed all queries in a suitable way.
